# The Effect of New Zealand Kanuka, Manuka and Clover Honeys on Bacterial Growth Dynamics and Cellular Morphology Varies According to the Species

**DOI:** 10.1371/journal.pone.0055898

**Published:** 2013-02-13

**Authors:** Jing Lu, Dee A. Carter, Lynne Turnbull, Douglas Rosendale, Duncan Hedderley, Jonathan Stephens, Swapna Gannabathula, Gregor Steinhorn, Ralf C. Schlothauer, Cynthia B. Whitchurch, Elizabeth J. Harry

**Affiliations:** 1 The ithree institute, University of Technology Sydney, Sydney, Australia; 2 School of Molecular Bioscience, University of Sydney, Sydney, Australia; 3 The New Zealand Institute for Plant and Food Research Ltd., Food Industry Science Centre, Palmerston North, New Zealand; 4 Comvita NZ Limited, Te Puke, New Zealand; 5 Department of Molecular Medicine & Pathology, Faculty of Medical and Health Sciences, The University of Auckland, Private Bag, Auckland, New Zealand; University of Padova, Medical School, Italy

## Abstract

Treatment of chronic wounds is becoming increasingly difficult due to antibiotic resistance. Complex natural products with antimicrobial activity, such as honey, are now under the spotlight as alternative treatments to antibiotics. Several studies have shown honey to have broad-spectrum antibacterial activity at concentrations present in honey dressings, and resistance to honey has not been attainable in the laboratory. However not all honeys are the same and few studies have used honey that is well defined both in geographic and chemical terms. Here we have used a range of concentrations of clover honey and a suite of manuka and kanuka honeys from known geographical locations, and for which the floral source and concentration of methylglyoxal and hydrogen peroxide potential were defined, to determine their effect on growth and cellular morphology of four bacteria: *Bacillus subtilis*, *Escherichia coli*, *Staphylococcus aureus* and *Pseudomonas aeruginosa*. While the general trend in effectiveness of growth inhibition was manuka>manuka-kanuka blend>kanuka>clover, the honeys had varying and diverse effects on the growth and cellular morphology of each bacterium, and each organism had a unique response profile to these honeys. *P. aeruginosa* showed a markedly different pattern of growth inhibition to the other three organisms when treated with sub-inhibitory concentrations of honey, being equally sensitive to all honeys, including clover, and the least sensitive to honey overall. While hydrogen peroxide potential contributed to the antibacterial activity of the manuka and kanuka honeys, it was never essential for complete growth inhibition. Cell morphology analysis also showed a varied and diverse set of responses to the honeys that included cell length changes, cell lysis, and alterations to DNA appearance. These changes are likely to reflect the different regulatory circuits of the organisms that are activated by the stress of honey treatment.

## Introduction

Wounds of the skin and mucosal layers can be generated by accidental trauma, surgery, maceration, inflammation and some cosmetic procedures (e.g. tattooing and piercing). For most superficial wounds, healing is prompt and requires no intervention. However, in some instances wounds can become infected, and in persons with impaired immunity or circulation, wounds can become non-healing, progressive and chronic. There is growing evidence that chronic wounds result from a complex interplay of host immunity and bacterial infection, and that infection can be due to a consortia of different species of bacteria embedded in a biofilm matrix that is highly resistant to antimicrobial therapy [1]. Planktonic bacteria are also important in chronic and acute wounds, and their release from biofilms has been proposed to maintain the inflammatory response within the wound [2], [3], as well as allowing seeding to other areas. The emergence of bacterial pathogens resistant to multiple antibiotics has exacerbated the problems associated with treating infected wounds, particularly in the hospital setting [4], [5]. There is an increasing need for new approaches to treat these infections, which are estimated to affect 6.5 million patients and to cost US$25 billion annually, with significant increases expected in the future [6].

Antimicrobial honey produced from the *Leptospermum scoparium* (manuka) plant from New Zealand has many features that make it a promising therapy for wound care. Manuka honey is broad in spectrum and able to inhibit a diverse range of bacterial and yeast pathogens, and is equally effective against multi-drug resistant bacteria [7]–[9]. This honey has been found to prevent the formation of biofilms and can disrupt pre-formed biofilms [10]–[11]. Resistance to manuka honey has never been observed and could not be attained under laboratory conditions that rapidly induced resistance to conventional antibiotics [7] [9]. And finally, honey stimulates the immune system and can promote wound healing [12]. There are a number of medicinal honey products on the market in the form of ointments, creams and impregnated gels. However their use in mainstream medicine remains limited [13].

Honey has several antibacterial features that are distinct from classical antibiotics, including high osmolarity, low pH, and the generation of hydrogen peroxide by the bee-derived enzyme glucose oxidase [14]. Some honeys also contain levels of bee defensin-1 that are sufficient to inhibit growth of bacteria [15], [16]. Active manuka honey contains high levels of the reactive dicarbonyl methylglyoxal (MGO) [17], [18], which forms non-enzymatically from nectar-derived dihydroxyacetone (DHA) during ripening. A diverse range of phenolics, complex carbohydrates and peptides have also been reported in honey samples, and these may contribute to or modulate antibacterial activity [19]–[20].

The antibacterial activity of honey is generally assessed by measuring the extent to which the indicator bacterium, *S. aureus*, is inhibited using agar diffusion or broth micro-dilution methods [21]. Similar tests have been performed to determine the inhibition of other bacterial and yeast species [7] [22], [23]. Manuka honey marketed for medicinal use generally uses a potency rating based on the “Unique Manuka Factor” (UMF), which measures antibacterial activity that is unrelated to the content of hydrogen peroxide, and is based on the *S. aureus* inhibition test. Alternatively, some medicinal honeys express potency as a direct assessment of MGO levels. While it has been established that manuka honey can inhibit the growth of bacterial cells, its effect on growth and cellular physiology among different bacterial pathogens, and how these change when the levels of the major antibacterial components, MGO and hydrogen peroxide, vary in natural honeys are unclear. These are important considerations for optimizing honey for wound care since sub-lethal levels of honey may have unanticipated effects, and there is emerging evidence that different organisms infecting a wound may respond quite differently to the active honey components [16] [23].

To address these issues this study set out to examine the growth response and cellular morphology of four different bacterial species, including two of the most common wound pathogens, to a suite of natural honey samples that differ in their levels of MGO and hydrogen peroxide production. This included samples of monofloral manuka honey with moderate to high MGO levels, samples of honey produced from the New Zealand kanuka *Kunzea ericoides* bush [19], where MGO levels are negligible but hydrogen peroxide is present, and manuka-kanuka blends that contain both active components at moderate levels. We included a set of controls to mimic the effects of sugar, to neutralize the effect of hydrogen peroxide, and to examine how MGO might act outside the honey milieu. We report here that while clinically relevant concentrations of honey are effective at inhibiting growth of all four bacteria, the growth and morphological responses at sub-lethal levels varied significantly between species. Furthermore, *P. aeruginosa* responded strikingly differently to the other three species (*B. subtilis*, *E. coli* and *S. aureus*). When present in sub-lethal concentrations, MGO extended the lag phase of bacterial growth in a dose-dependent manner, and the organisms eventually resumed normal growth, presumably by detoxifying the MGO. Topical wound dressings should therefore contain a high level of active honey to ensure wound pathogens are eliminated.

## Materials and Methods

### Honey Samples

Table 1 lists the New Zealand honey samples used in this study, which included monofloral manuka (M1, M2, M3), monofloral kanuka (K1 and K2), manuka-kanuka blends (MK1, MK2, MK3, MK4) and clover (C) honey. Samples were chosen based on their levels of methylglyoxal (MGO; previously reported in Stephens *et al.* 2010), and hydrogen peroxide, determined in this study. Manuka, kanuka and manuka-kanuka honey samples were supplied by Comvita New Zealand Ltd. (Te Puke, New Zealand) and the clover honey sample was a commercially-packaged New Zealand white clover honey [19]. Native New Zealand honey is produced by bees foraging in their local environment and cannot be guaranteed to be 100% monofloral, however the supplied samples were considered to be as representative of pure honey from a single floral origin as possible. Details of other chemical components in the manuka and kanuka honeys have been described previously [19]. All samples were stored in the dark at 4°C and were diluted fresh for use in all assays. All honey concentrations are expressed as % w/v.

**Table 1:** Floral source, MGO and H_2_O_2_ Levels of Honeys.

Code	Previous code^a^	Honey	Floral source	Antibacterials	
				MGO^b^_(mg/kg)_	H_2_O_2_ c_ (mM/h)_
M1	2	Manuka^d^	*Leptospermum scoparium var incanum*	651.4	0.532
M2	13	Manuka e	*L. scoparium var incanum+Kunzea (?)*	1004.3	0.282
M3	7	Manuka^e^	*L. scoparium var incanum*	1541.3	0.239
K1	22	Kanuka^e^	*Kunzea ericoides*	5.6	0.360
K2	21	Kanuka^e^	*Kunzea ericoides*	37.1	0.327
MK1	23	Manuka-Kanuka^e^	*Kunzea ericoides+manuka (?)*	173.6	0.583
MK2	–	Manuka-Kanuka	*Kunzea ericoides+manuka (v. likely)*	229.8	0.448
MK3	18	Manuka-Kanuka^e^	*L. scoparium var 'triketone'+Kunzea*	269.9	0.345
MK4	15	Manuka-Kanuka^d^	*L. scoparium var 'triketone'*	307.8	0.380
C	24	Clover^f^	*Trifollium spp.*	trace	0.029

a As reported in Stephens *et al.* (2010).

b MGO (methylglyoxal) levels, reported in Stephens *et al.* (2010).

c H_2_O_2_ (hydrogen peroxide) levels are expressed as mean H_2_O_2_ production rate in 1 mL of 10% w/v honey.

d Samples collected from hive sites.

e Aged samples from drums supplied by apiarists and purchased as designated type.

f Obtained commercially.

### Hydrogen Peroxide Assay

The level of hydrogen peroxide produced by the honey samples was determined using a hydrogen peroxide/peroxidase assay kit (Amplex Red, Molecular Probes, Life Technologies Corp., Carlsbad, CA, USA). The assay, which measures the oxidation by hydrogen peroxide of the non-fluorescent substrate Amplex Red to highly fluorescent resorufin [24], was conducted in 96-well microtitre plates according to the manufacturer’s instructions. Resorufin fluorescence was measured at 530 nm excitation/590 nm emission using a SpectraMax Gemini EM (Molecular Devices, LLC, Sunnyvale, CA, USA) fluorometer. Hydrogen peroxide standards from 5–20 µM were used to produce a standard curve, which was then used to assess production in duplicate samples of 2.5% and 5% w/v dilutions of the honey samples. The results were normalized to mM H_2_O_2_/h in 1 mL of 10% w/v honey solution.

### Bacterial Strains and Growth Media

Four different bacterial species were examined: the Gram-positive bacteria *B. subtilis* 168 [25] and *S. aureus* ATCC 25923 (American Type Culture Collection), and the Gram-negative bacteria *E. coli* O157:H7 [26] and *P. aeruginosa* PAO1 (ATCC 15692). *B. subtilis* is a well-studied model organism, and the other three species are clinically relevant pathogens. Growth media were selected to allow optimal growth of the different bacterial species: Luria-Bertani (LB) (Oxoid Ltd., Basingstoke, Hampshire, UK) broth and agar were used for *E. coli, P. aeruginosa* and *B. subtilis*, while Tryptone Soya Broth and agar (Oxoid Ltd., Basingstoke, Hampshire, UK) was used for *S. aureus*.

### Growth of Bacterial Cultures

Planktonic bacteria in wounds, while viable, are likely to be growing very slowly, if at all. We therefore added honey to diluted stationary-phase bacterial cultures so that it would more accurately represent the addition of a honey dressing to a chronic wound. Single colonies of bacteria grown on agar were used to inoculate broth cultures. These were grown overnight at 37°C on an orbital shaker at 250 rpm (Bioline™, Australia) except *B. subtilis,* which was grown overnight at 30°C with slower shaking using a gyrotory waterbath shaker (New Brunswick Scientific, Enfield, CT, USA). The slower shaking conditions for *B. subtilis* ensure that this culture does not spend too long in stationary phase, which would delay entry into exponential growth upon dilution. Cell density of the overnight cultures was assessed using serial-dilution plating and was approximately 10^9^ colony-forming units (CFU)/mL. A suspension from the overnight culture was then diluted to a cell density of 10^3^ CFU/mL in fresh media containing honey to give a final volume of 150 µL. For each growth assay, a freshly prepared 50% (w/v) honey stock solution was made by weighing the appropriate amount of honey and mixing this with an equivalent amount of sterilized distilled water. This stock solution was then further diluted with the appropriate growth medium to give the required honey concentration. Growth of each bacterial species was tested in six concentrations of each honey (1%, 2%, 4%, 8%, 16% and 32% w/v) in a 96-well microtitre plate format. A microtitre plate reader (Biotek PowerWave HT®, Bioteck Instrunents Inc, Winooski, VT, USA) programmed to measure the optical density hourly at 595 nm (OD_595nm_) (Gen5®, BioTek) was used to assay cell growth over 24 hours at 37°C, with moderate shaking (1800 rpm, amp. 0.549 mm x-axis). Two biological replicates, each with four technical replicates were performed for the growth assays and each growth curve produced in Figure S1 represents the average of all data. Growth curves were presented using GraphPad PrismV. 5.0c (Graphpad Software, San Diego, CA, USA).

A comprehensive range of control treatments was included for each organism in the microtitre-plate growth assays. These included: (i) a no-treatment control; (ii) a sugar solution comprising 45% glucose, 48% fructose and 1% sucrose, (diluted as above for honey) to identify any effects on bacterial growth due to the high sugar content in honey; (iii) honey plus catalase (1 mg/mL) to neutralize hydrogen peroxide [14]; (iv) a catalase-only control; (v) MGO diluted in water to concentrations similar to those present in honeys M1, M2 and M3 (600, 1,000 & 1,500 mg/kg undiluted honey) at the various tested concentrations, to assess the effect of MGO alone on bacterial growth; vi) the same MGO dilutions plus catalase; and (vii) MGO diluted in sugar solution to the same concentrations as above and with added catalase. MGO was obtained as a 40% solution in water (Sigma-Aldrich Co., St Louis, MO, USA).

### Growth Curve Data Analysis

Initial inspection of the bacterial growth data indicated that the consistent major effect of honey on growth dynamics was an extended lag phase, such that entry into exponential growth was delayed, and this increased with increasing honey concentrations. Thus, we focused on how honey altered the duration of lag phase. Lag phase was calculated as the period from inoculation to onset of log phase, or to 10% of maximal culture absorbance. Given the large number of different growth curves (128 individual growth assays with 6 different honey concentrations per assay), we automated the calculation of these parameters by fitting the absorbance values from the bacterial growth experiments to a generalized logistic curve (equation 1), a sigmoid function used for growth modeling, using the Genstat program (Release 11.1 (PC/Windows) 28 January 2011, VSN International Ltd, UK). Due to variable T values, this generalized logistic curve fitted better than a corresponding Gompertz curve (not shown).
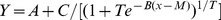
(1)


Here, A = the lower asymptote; C = the upper asymptote; M = time of maximum growth; B = growth rate, and T = time near which maximum (stationary phase) growth occurs.

With these parameters, we were able to compare the effect of the different honey samples on growth simply by plotting the duration of lag phase (time (h)) in the presence of varying honey concentrations (% w/v). This conversion from growth curve to lag phase duration is illustrated in Figure S2, where a sample curve of *E. coli* growth in response to a series of manuka honey M3 dilutions (Fig. S2A, onset of log phase or 10% of maximal culture absorbance at each honey concentration shown by “x”) is converted to the corresponding lag-phase honey dose response (Fig. S2B).

In the vast majority of cases when growth of a culture was detected by absorbance measurement, the maximal culture absorbance was very similar to the no-honey control culture. However in a few cases the maximal absorbance of the treated culture was less than 10% of the maximal culture absorbance of the no-honey control. In these cases, it was assessed as ‘no growth’ over the 24-hour period.

### Cell Staining and Microscopy

Bacterial cultures treated with either 4% (w/v) honey M3 or honey MK1 (Table 1) were harvested from samples obtained from the middle of the prolonged lag phase induced by honey treatments, and at log phase (which we will refer to as log phase) when cultures had resumed apparently normal growth. If a prolonged lag phase was not observed, lag phase cells were obtained from within the first half hour of incubation. Untreated cells from lag and log phases of growth were also harvested for analysis, with the lag-phase cells collected 30 min after inoculation as described above. Harvested cells were treated for microscopy as described previously [27] but with the following modifications: 20 µL of fixed cells were diluted 1∶1 with the DNA staining agent DAPI (4',6-diamidino-2-phenylindole; Life Technologies), to give a final DAPI concentration of 0.4 µg/mL for *E. coli*, *B. subtilis* and *P. aeruginosa*, and 0.8 µg/mL for *S. aureus*. Triplicate 10 µL aliquots of the stained cells were then placed in separate wells of a multi-well microscope slide (MP Biomedicals, LLC, Eschewege, Germany) that had been treated with 0.01% poly-L-lysine (Electron Microscopy Sciences, Hatfield, PA, USA). After 15 min at room temperature, the liquid was removed and 50% glycerol was placed on each sample. A coverslip was then placed on all samples and the edges of the coverslip were sealed with nail polish.

Cells were imaged using phase-contrast and fluorescence microscopy with a Zeiss Axioplan 2 fluorescence microscope equipped with a Plan ApoChromat (100x, NA 1.4; Carl Zeiss AG, Oberkochen, Germany) objective lens, and images were captured using a Zeiss AxioCam MRm cooled CCD camera controlled by AxioVision software (version 4.5; Carl Zeiss). Fluorescence microscopy to visualize DNA stained with DAPI used a 100 W high pressure mercury lamp passed through filter set 02 (Carl Zeiss) as a light source. Image processing was performed using AxioVision software version 4.5 (Carl Zeiss).

### Image Data Analysis

Cell length, cell lysis and DAPI staining were assessed by digital analysis of the captured images. Cell length and DAPI staining were scored only for unlysed cells. A total of 152 fields of cells were imaged and analyzed. Cell length (or diameter in the case of *S. aureus*) was measured using MicrobeTracker (version 0.929) [28]. We used this MATLAB-based software to detect and outline bacterial cells in the microscopy images and measure cell lengths automatically. The optimized parameters (incorporated into the MicrobeTracker software) included a modification to algorithm 4 to enable accurate cell length measurements of rod-shaped organisms in the case of *E. coli*, *B. subtilis* and *P. aeruginosa.* For *S. aureus* algorithm 1 was optimized to enable measurement of the size of these spherical cells [28]. The individual cell length information was then extracted and statistical analysis was performed in GraphPad Prism. One-way ANOVA and Tukey’s multiple comparison tests were performed with the no-honey treated cells as controls. Cells that appeared lysed due to changes in their contrast under phase-contrast microscopy were scored and cell lysis was expressed as a percentage of the whole population. Only cells that remained intact but appeared to lose their cytoplasmic contents were scored, thus underestimation of cell lysis was possible, but this was consistent across all samples. For all experiments, at least 100 cells were scored, except for M3-treated *B. subtilis* and *S. aureus* cells, where at least 50 cells were scored.

## Results

### Growth Responses to Honey, MGO, Sugar and Catalase

The growth response of two Gram-positive bacteria, *B. subtilis* 168 and *S. aureus* ATCC 25923, and two Gram-negative bacteria, *E. coli* O157:H7 and *P. aeruginosa* PAO1 (ATCC 15692) to the 10 honeys and various control solutions were assessed. These data comprised nearly 900 growth curves in 128 graphs (Fig. S1). For the honey treatments each graph represents a particular honey at six concentrations with a single organism as well as a no-honey control, which was carried out alongside each honey sample on the multi-well plates. A comprehensive range of control treatments were included in the growth assays to determine the effect of various honey components on growth of the four bacteria (see Materials and Methods).

**Figure 1:**
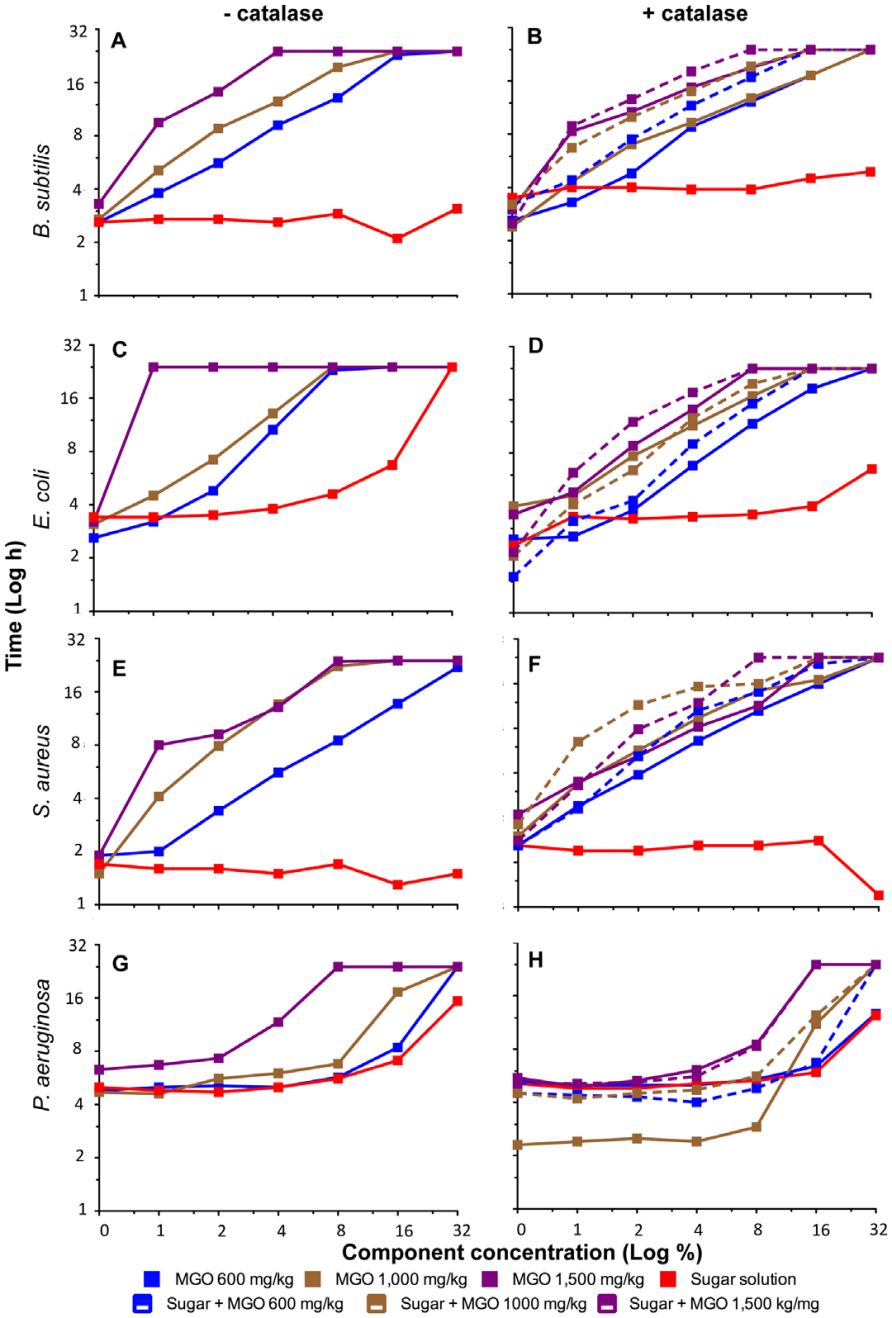
Effect of sugar, MGO and catalase on growth of bacteria. Overnight cultures of *B. subtilis, E. coli, S. aureus* and *P. aeruginosa* were treated with various components, including catalase, MGO, sugar, and a combination of MGO and sugar at various concentrations equivalent to honeys at the corresponding concentrations shown on the x-axis. The MGO/sugar experiments were performed in the absence (left-hand graphs) and presence (right-hand graphs) of catalase as indicated. The MGO levels correspond to honeys M1 (651.4 mg/kg MGO), M2 (1004.3 mg/kg MGO) and M3 (1541.3 mg/kg MGO) at 1%–32% (w/v). Optical density was recorded at 595 nm every hour for 24 hours. For each component concentration, the time it takes for the culture to reach log phase (assessed as at least 10% of the final culture absorbance of the untreated culture) is plotted on the x-axis. The derivation of this value is described in Materials and Methods. A value of 24 hours on the y-axis denotes ‘no growth’. An untreated control was performed alongside each particular treatment, and the starting OD_595_ (zero time-point on x-axis) is plotted for that particular honey experiment.

**Figure 2:**
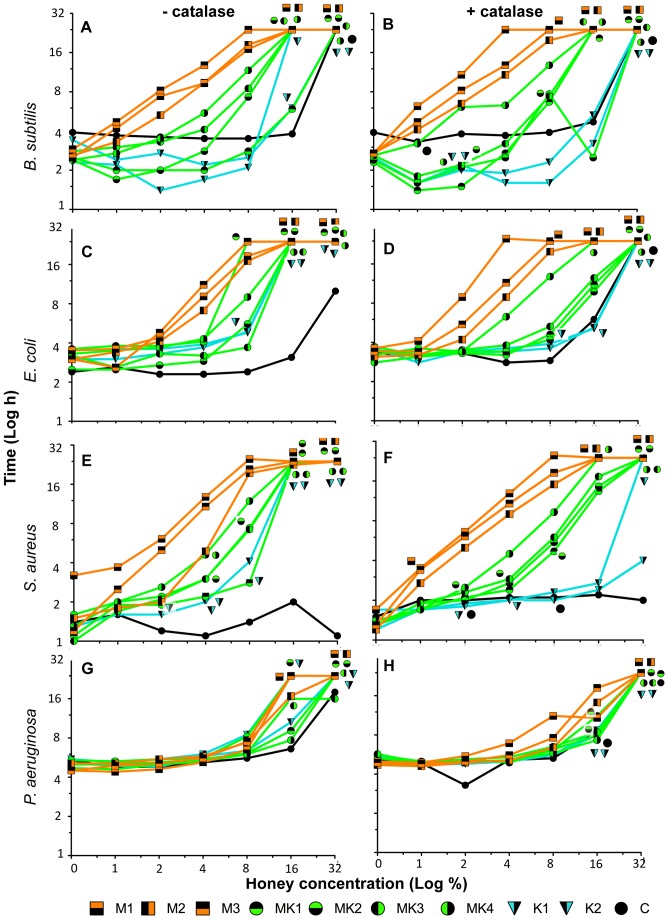
Effect of New Zealand manuka, kanuka and manuka-kanuka blended honeys on bacterial growth. Overnight cultures of *B. subtilis, E. coli, S. aureus* and *P. aeruginosa* were treated with ten different honeys, plus or minus catalase: three manuka honeys, M1, M2 and M3; two kanuka honeys, K1 and K2; four manuka-kanuka blended honeys, MK1, MK2, MK3 and MK4; and one clover honey, C, at various concentrations (from 1%–32% (w/v), increasing in 2-fold series). Optical density was recorded at 595 nm every hour for 24 hours. For each honey concentration, the time it takes for the culture to reach log phase (assessed as at least 10% of the final culture absorbance of the untreated culture) is plotted on the x-axis. The derivation of this value is described in Materials and Methods. A value of 24 hours on the y-axis denotes ‘no growth’. Where symbols for a particular honey overlap, we have surrounded the point on the graph by all the symbols relevant to that point. This occurs in several cases for 16% and 32% honey treatments. An untreated control was also performed alongside each particular honey treatment, and the starting OD_595_ (zero time-point on x-axis) is plotted for that particular honey experiment.

Time spent in lag phase before entry into exponential growth emerged as the most notable difference among bacteria in their response to the different honey types (see Materials and Methods). We therefore focused our analysis on growth inhibition on this parameter, expressed as the time taken (in hours) for the bacterial culture to reach 10% maximal culture absorbance. The graphs presented in Figure 1 and Figure 2 summarize the growth responses of the four different organisms to the control solutions (Fig. 1) and the honeys (Fig. 2). In these graphs, the time (h) taken for a culture to enter logarithmic growth (measured as at least 10% maximal culture absorbance; y-axis) is plotted against the honey (or component) concentration (x-axis) for each organism both in the absence (left panel) and presence (right panel) of catalase. Note that the faster the rise of the line, the longer the cells are arrested in lag phase at lower honey concentrations, and hence the more effective a particular honey is at inhibiting the growth of that organism. Culture growth was monitored over 24 hours, and if no growth occurred over 24 hours, it is referred to as ‘no growth’ or complete inhibition.

The starting absorbance values differ in each case in Figures 1 and 2 because for each honey, the no-honey control was included to more accurately reflect the experimental conditions during that particular experiment.

### Growth Dynamics in Response to Controls: MGO and Sugar in the Presence and Absence of Catalase

The addition of catalase to an overnight culture of bacterial cells had essentially no effect on the duration of the lag phase, or any other aspect of growth of the four organisms when compared with an untreated control culture (Fig. S1). Sugar alone had a small but variable effect on the growth of the bacteria: the Gram-positive species *B. subtilis* and *S. aureus* were unaffected even at high concentrations, while the two Gram-negatives became inhibited at 16–32% (Fig. 1A, 1C, 1E, 1G).

The addition of MGO at 0–32% of the concentrations present in manuka honey samples M1, M2 and M3 (starting concentrations of 600, 1,000 and 1,500 mg/kg honey) generally showed a dose-dependent extension of lag phase (Fig 1A, 1C, 1E, 1G). This was most severe for *E. coli*, followed by *B. subtilis* and *S. aureus*, and was lowest in *P. aeruginosa*. The difference in sensitivity to MGO alone between the organisms was most obvious at the lowest MGO concentration used (blue lines in Fig. 1A, 1C, 1E, 1G).

The addition of catalase to the MGO treatments shortened the lag-phase extension in most cases, so that the onset of log phase occurred earlier. With the exception of *P. aeruginosa,* the bacteria were still increasingly sensitive to increasing MGO concentrations in the presence of catalase (Fig. 1, compare left with right panels). The addition of sugar to MGO in the presence of catalase had a small but noticeable effect on delaying the onset of log phase further for all organisms except *P. aeruginosa*, particularly at the lower sugar concentrations tested (equivalent to ≤8% honey; Fig. 1).

### Growth Response in the Presence of Natural Honeys

Graphs summarizing the effect of the different honey types on growth of the four bacterial species are shown in Figure 2. From these, four key features are particularly apparent: first, there is a general trend of greater growth inhibition by honeys containing more MGO, with M>MK>K>clover honey; second, the addition of catalase causes a shift of the curves to the right for most honey types indicating a rescue of growth inhibition; third, *P. aeruginosa* has a completely different pattern of growth inhibition compared to *B. subtilis*, *E. coli* and *S. aureus*; and fourth, the effect of clover honey is different for the different bacteria. These points will be explored further below, where the response of the bacteria to each honey type is described.

#### Manuka honey

Manuka honey samples M1, M2 and M3 (Table 1) have the highest MGO concentrations of the honeys tested, at 651.4, 1004.3 and 1541.3 mg/kg honey, respectively. These three honeys were the most effective in inhibiting growth of *B. subtilis*, *E. coli* and *S. aureus* and all resulted in similar levels of growth inhibition. Low honey concentrations (1–4%) caused significant lag-phase extension and growth was completely inhibited once concentrations reached 8–16%.

In the presence of catalase the monofloral manuka honeys remained the most effective of the natural honeys at inhibiting growth of *B. subtilis*, *E. coli* and *S. aureus*, indicating that the non-peroxide component/s in these honeys is the over-riding component responsible for their high levels of growth inhibition.

In contrast to *B. subtilis*, *E. coli* and *S. aureus*, there was very little or no lag-phase extension when *P. aeruginosa* was treated with low concentrations of honeys M1–M3, and complete inhibition only occurred at 16% of honeys M3 and M1 and 32% of honey M2. *P. aeruginosa* was relatively sensitive to sugar (Fig. 1G), which likely accounts for some of the inhibition. The addition of catalase increased the concentration of honeys M1 and M3 required for complete growth inhibition 2-fold (to 32%). These data and those shown in Figure 2 indicate that *P. aeruginosa* is relatively insensitive to both hydrogen peroxide and MGO, and that at 32% manuka honey inhibition can be attributed to non-peroxide component/s.

#### Kanuka honey

The kanuka honeys, K1 and K2, had very low levels of MGO (5.6 and 37.1 mg/kg, respectively), but moderate rates of hydrogen peroxide production (0.360 and 0.327 mM/h, respectively) compared to the other honeys tested. At low concentrations (1–8%), and particularly in the presence of catalase, K1 and K2 were amongst the least effective of the honeys at inhibiting growth of *B. subtilis*, *E. coli* and *S. aureus*, with very little or no lag-phase extension compared to the no-honey cultures. Complete growth inhibition with K1 and K2 occurred at 16% or 32%.

Although the addition of catalase to K1 and K2 made them less effective at inhibiting growth of *B. subtilis*, *E. coli* and *S. aureus*, complete growth inhibition still occurred at 32%; the exception being honey K1, where lag phase was only extended by 4 hours (Fig. 2F). The non-peroxide component causing this growth inhibition is not likely to be due to MGO, or at least MGO acting alone, since the amount present in 32% K1 and K2 is 11.8 and 1.8 mg/kg respectively, which is equivalent to 1% of 660 mg/kg MGO and is therefore too low to affect growth of these bacteria (Fig. 1A, 1C, 1E; light blue line). The inability of honey K1 to inhibit the growth of *S. aureus* suggests that the component/s that contribute to complete growth inhibition of *B. subtilis* and *E. coli* are not active against *S. aureus*. Alternatively there may be a component in honey that is specifically active against *S. aureus* but requires hydrogen peroxide for its production and/or activity. Note that *S. aureus* was also the only species that was not inhibited by clover honey (see below).

*P. aeruginosa* growth was completely inhibited by kanuka honeys at 16% (K1) and 32% (K2), and very little lag-phase extension was observed. Catalase addition rescued this effect to some extent, but at 32% the kanuka honeys completely inhibited growth of *P. aeruginosa*. Again, this suggests component/s additional to MGO or peroxide are present in these honeys that affect growth of this organism. The most striking observation for *P. aeruginosa* that was distinctly different from the other organisms was that growth was similarly affected by kanuka honeys as by manuka honeys.

#### Manuka-kanuka honey blends

The responses of bacteria to the manuka-kanuka honey blends, designated MK1–MK4, are shown in green in Figure 2. These honeys have intermediate levels of MGO (ranging from 173.6–307.8 mg/kg) that are between those of the pure manuka and kanuka honeys, and variable but significant levels of hydrogen peroxide (Table 1). Treatment with these honeys gave a level of inhibition that was generally between that of the pure manuka and pure kanuka honeys, especially when the hydrogen peroxide was removed by catalase. In addition, the degree of growth inhibition related largely to the level of MGO, with MK4, which has the highest level of MGO of the blended honeys (Table 1), normally being the most effective at inhibiting growth.

While the overall pattern of growth inhibition of *B. subtilis*, *E. coli* and *S. aureus* by the MK honeys was similar, there were some notable differences in how *E. coli* responded to the different blends. In the absence of catalase MK1 inhibited *E. coli* growth to a similar extent as the manuka honeys, with complete growth inhibition at 8%. MK1 has a low level of MGO (173.6 mg/kg) compared to the manuka honeys but has the highest hydrogen peroxide production rate of all honeys. Catalase addition to MK1 reduced the level of growth inhibition for *E. coli* to a level well below that of all three manukas. These observations suggest that *E. coli* growth can be maximally inhibited by honeys that either have a high level of hydrogen peroxide production or have high levels of MGO.

*P. aeruginosa* displayed little or no lag-phase extension or growth inhibition for any of the blended honeys until concentrations reached 16% or 32%. Overall, there was no clear trend in how *P. aeruginosa* responded to the varying levels of MGO and hydrogen peroxide in the different blends, however complete growth inhibition was achieved at 32% in the presence of catalase, indicating that the inhibition does not require hydrogen peroxide.

#### Clover honey

The clover honey sample had no detectable MGO and almost no hydrogen peroxide production (0.029 mM H_2_O_2_/h; Table 1). Up to 16% clover honey had little effect on the growth of the four organisms (Fig. 2). At 32%, *S. aureus* growth remained unaffected, while the two Gram-negative species, *E. coli* and *P. aeruginosa*, showed a significant lag-phase extension. This is commensurate with the response of these two organisms to 32% sugar (Fig. 1A, 1C, 1E and 1G). However, while the addition of catalase to clover honey slightly increased lag phase extension, this was not seen for the corresponding sugar control. Interestingly, 32% clover honey completely inhibited growth of *B. subtilis*, both in the presence and absence of catalase even though sugar alone at equivalent concentrations had no effect on the growth of this organism. This suggests the presence of one or more components in clover honey to which *B. subtilis* growth is particularly sensitive.

#### Other observations not fitting growth inhibition trends

Although there were clear trends in growth inhibition in response to treatment with honeys and control solutions discussed above, there were certain observations that did not fit these trends that are worth acknowledging. This includes: M1, which has the lowest level of MGO, was the most active manuka honey for *B. subtilis, E. coli* and *S. aureus* in the presence of catalase (Fig. 2B, 2D, 2F); the apparent abrupt (and reproducible) decrease in growth inhibition of the MK2 honey against *B. subtilis* at 16% in the presence of catalase (Fig. 2B); the incomplete inhibition of *P. aeruginosa* by honey MK3 only observed when catalase was not present (Fig. 2G); and a higher level of inhibition of *E. coli* by clover honey in the presence of catalase (Fig. 2D). Given the complexity of honey it is likely that the growth inhibition we observe in these analyses cannot always be solely accounted for by the presence MGO and hydrogen peroxide, and other components may exert independent action or may modulate the response of bacteria to MGO- and hydrogen peroxide-based toxicity.

### Cellular Morphology Response in the Presence of Natural Honeys

To determine morphological changes that occur in response to honey containing relatively high levels of MGO or hydrogen peroxide, *B. subtilis*, *E. coli*, *S. aureus* and *P. aeruginosa* were exposed to honey samples M3 (highest MGO with lowest hydrogen peroxide production of the tested honeys) and MK1 (highest rate of hydrogen peroxide and relatively low MGO; Table 1). Cells were treated with 4% (w/v) of each honey, which is the highest concentration that still allowed growth of all four bacteria (see above; Fig. 2). Cell morphology was analysed during lag- and log-phase growth and included measures of cell shape changes (length or width), cell lysis (breakage of cells or leakage of cytoplasm indicating cell envelope or growth abnormalities), and detection of chromosomal DNA abnormalities by DAPI staining.

#### High-level MGO honey and cell morphology

**Figure 3:**
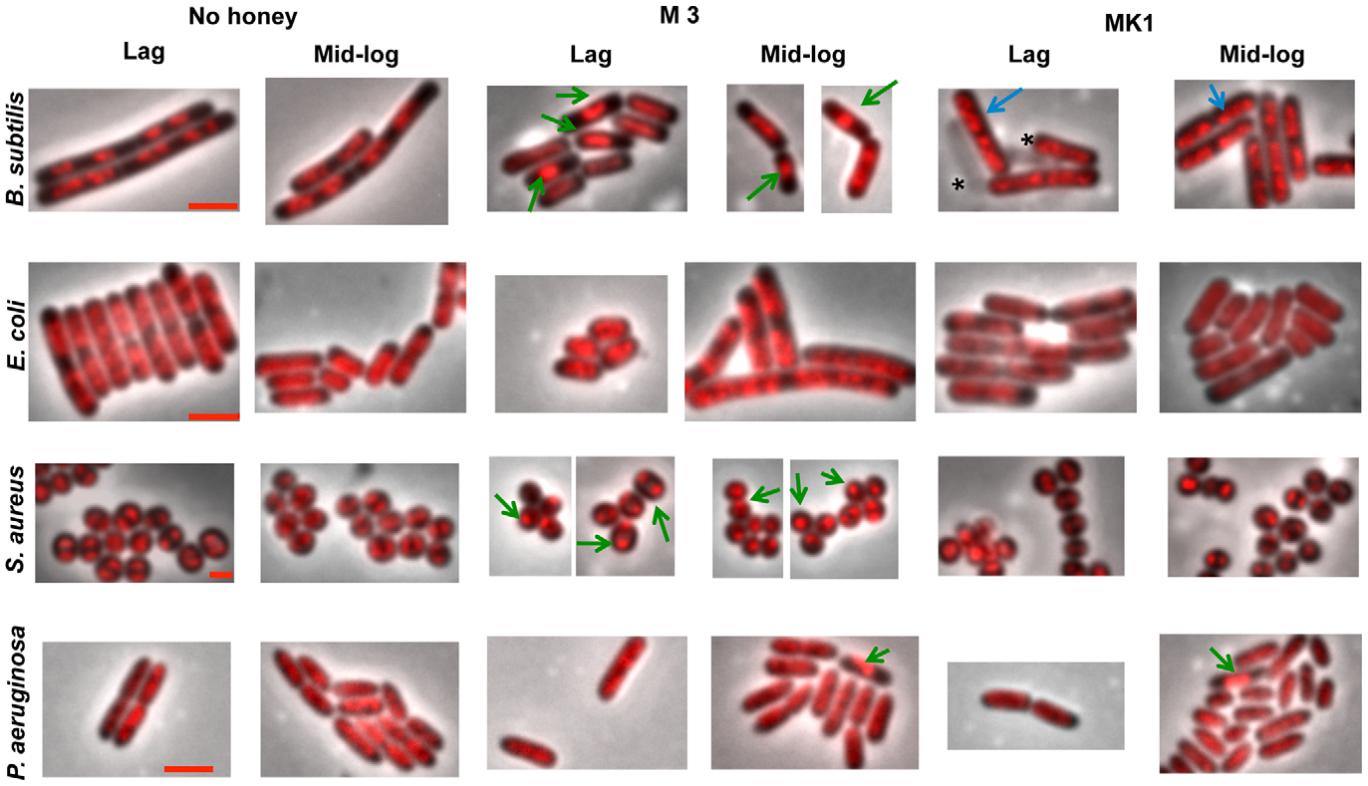
Cellular morphology of bacterial cells treated with a high-MGO honey and a high-hydrogen peroxide honey. The effects of 4% (w/v) of a high-MGO honey (M3) and a high-hydrogen peroxide honey (MK1) on bacterial cellular morphology were examined. Overnight cultures of *B. subtilis, E. coli, S. aureus* and *P. aeruginosa* were treated with these honeys, cells collected at both lag and log phases of growth as indicated in Figure S2, fixed with glutaraldehyde, stained with DAPI and imaged using fluorescence microscopy. All images are overlays of the phase-contrast image and the DAPI-stained (red) fluorescence image. The two left-hand panels show the no-honey treated control cells, the two middle panels M3 honey-treated cells, and the two right-hand panels show the MK1 honey-treated cells. In all images, condensed DNA is shown by green arrows; and dispersed DNA in *B. subtilis* cells is shown by blue arrows. An asterisk indicates lysed cells for *B. subitlis* (MK1, lag-phase cells). The scale bar represents 2 µm, except for *S. aureus* images, where it represents 1 µm.

Treatment with honey M3 induced an extended lag phase for all bacterial cultures except *P. aeruginosa* (Fig. S1; Fig. 2). Morphological changes are shown in Figure 3 and charted in Table 2, and mean cell lengths are recorded in Table S1. During the extended lag phase (or the initial lag phase for *P. aeruginosa*), cells of *B. subtilis*, *E. coli* and *S. aureus* were significantly shorter (p<0.05) than untreated cells, while *P. aeruginosa* cells were longer (Table 2; Fig. 3). In addition, a significant percentage of the shorter cells of *B. subtilis* (29%) and *S. aureus* (57%) had a condensed chromosome (green arrows in Fig. 3). In the *B. subtilis* cells only one bright region of DAPI staining occurred instead of the characteristic two regions that represent replicating chromosomes (Fig. 3; Table 2). Likewise the *S. aureus* cells with condensed chromosomes showed one or two very small spots of DAPI-stained DNA, unlike the two larger lobes of DNA that represent replicating chromosomes in the no-honey control cells. No changes to DNA appearance under these conditions were observed for *E. coli* or *P. aeruginosa*.

**Table 2:** Cell morphology changes with high-MGO honey and high-hydrogen peroxide honey treatment^a^.

	*B. subtilis*			*E. coli*			*S. aureus*			*P. aeruginosa*		
	Length	Lysis	DNA	Length	Lysis	DNA	Length	Lysis	DNA	Length	Lysis	DNA
**M3 lag**	↓ (1.8x)	–	Condensed (29%)	**↓** (1.8x)	–	–	**↓** (1.2x)	–	Condensed (57%)	**↑** (1.1x)	–	–
**M3 log**	**↓** (1.4x)	–	Condensed (23%)	**↑** (1.6x)	–	–	**↓** (1.2x)	–	Condensed (57%)	**↓** (1.1x)	–	Condensed (2%)
**MK1 lag**	**↓** (1.2x)	34%	Dispersed (90%)	–	–	–	**↓** (1.1x)	–	–	–	–	–
**MK1 log**	**↓** (1.2x)	2%	Dispersed (99%)	–	–	–	–	–	–	**↓** (1.6x)	–	Condensed (2%)

a Actual mean cell lengths and statistics are shown in Table S1.

**↓**Statistically significant decrease compared to no-honey treated cells (*p<0.05*).

**↑**Statistically significant increase compared to no-honey treated cells (*p<0.05*).

–No change.

Following entry into log phase, cells treated with M3 honey were still significantly different to untreated cultures (Table S1 and Fig. 3). *B. subtilis* and *S. aureus* cells remained shorter, to a similar degree observed in lag phase, and chromosomes remain condensed. *E. coli* cells became significantly longer than their untreated counterparts, while *P. aeruginosa* cells became slightly but significantly shorter (Table 2). In addition, 2% of the *P. aeruginosa* cell population now showed a condensed chromosome by DAPI staining (green arrows in Fig. 3; Table 2).

In summary, treatment with 4% M3 honey changed mean cells lengths of all four populations of bacteria in both lag and log phases of growth, but the direction and the extent of change varied. The greatest changes to cell length were observed with *B. subtilis* and *E. coli* (Table 2). Only the Gram-positive organisms had condensed DNA for both growth phases.

#### High-level hydrogen peroxide honey and cell morphology

Treatment with 4% MK1 honey did not result in an extended lag phase for any organism, however this honey was particularly inhibitory to *E. coli* (Fig. 2). The most significant changes to cell morphology induced by MK1 during the initial stages of growth were observed in *B. subtilis*, where cells were on average significantly shorter, as was seen with M3 but to a lesser extent. A significant proportion of cells (34%) appeared lysed as judged by a decrease in contrast visualized using phase-contrast microscopy (asterisks in Fig. 3; Tables S1 and 2), and the DNA in the vast majority of unlysed cells (90%) appeared dispersed compared to untreated cells (Fig. 3; Table 2). No morphological changes were observed in *E. coli* or *P. aeruginosa* cultures, and the only change to *S. aureus* cells was a slight but significant decrease in cell diameter (Table 2).

In log-phase MK1-treated *B. subtilis* cultures, the extent of cell lysis was reduced from 34% in the lag-phase cultures to 2%. However, cells were still shorter on average compared to control cells (Table 2 and Table S1; Fig. 3), and the frequency of cells with a dispersed DNA appearance remained very high (99%). Log-phase *S. aureus* cells showed a normal morphology (Fig. 3; Table 2), while *P. aeruginosa* cells were significantly shorter (Fig. 3; Table S1 and 2). Two percent of the log-phase MK1-treated *P. aeruginosa* cells had the same condensed chromosome phenotype seen in the log-phase M3-treated *P. aeruginosa* cells. *E. coli* log-phase cells remained similar in appearance to the control cells (Table 2).

In summary, MK1 honey caused less alteration to cellular morphology than the high-MGO honey, M3. *B. subtilis* cells were the most significantly affected, with dispersed DNA, cell lysis and cell length changes. *E. coli* and *S. aureus* had little or no apparent change. *P. aeruginosa* cells were shorter in log phase only.

### Correlation of Growth Inhibition and Morphological Changes Induced by Honey

A summary of combined growth and morphology data is given in Table 3. Overall this shows that MGO and manuka honeys are the most effective at inhibiting growth of all organisms except *P. aeruginosa*, followed by manuka-kanuka blended honeys, kanuka honeys and then clover. *P. aeruginosa* is much less sensitive to the honeys compared to the other three organisms, with little difference in growth inhibition by the different honeys, including clover. *B. subtilis* shows more morphological changes than the other bacteria for both the high-MGO and the high-hydrogen peroxide honeys, followed by *S. aureus*, *P. aeruginosa* and then *E. coli*. Thus, with the exception of *B. subtilis,* where both growth and morphology are profoundly affected by honey, the number and severity of morphological changes do not link clearly to the level of MGO or hydrogen peroxide in the honey, or to the effectiveness of the honey to inhibit growth. *E. coli* had very little apparent morphological changes even though its growth was affected in a similar way to *B. subtilis* and *S. aureus* and to a much greater extent than *P. aeruginosa*, with the latter conversely showing more profound morphological changes.

**Table 3:** Summary of growth and morphological effects of honeys and control treatments on all organisms.

	Growth Inhibition						Cell Morphology^a^	
Organism	MGO	Sugar	Clover	M	K	MK	High-MGO Honey (M3)	High-H_2_O_2_ Honey (MK1)
***B. subtilis***	XXX^b^	–c	X	XXXX	XX	XXX	Shorter cells; condensed DNA(25%)	Shorter cells; lysis; dispersed DNA (95%)
***E. coli***	XXXX	XX	X	XXXX	XX	XX	Shorter and longer cells	–
***S. aureus***	XXX	–	–	XXXX	XX	XX	Shorter cells; condensed DNA(57%)	Shorter cells
***P. aeruginosa***	XX	X	X	XX	XX	XX	Shorter and longer cells;condensed DNA (2%)	Shorter cells; condensed DNA (2%)

a this data includes data from both the log and lag phases of growth.

b The number of crosses increases the more growth is inhibited.

c Means no effect.

## Discussion

With the dearth of development of new classes of antibiotics to treat infections caused by resistant organisms, honey is increasingly valued for its broad-spectrum antibacterial activity and effectiveness as a treatment for chronic wound infections. However, as with all natural products, there is significant chemical variation between different honey preparations [19] and this is likely to affect the level of antibacterial activity, and possibly, treatment outcomes. In this study, we have therefore assessed a series of geographically- and chemically-defined New Zealand manuka, kanuka and manuka-kanuka blended honeys with varying concentrations of MGO and hydrogen peroxide to determine their effectiveness in inhibiting the growth of different species of bacteria. We show here that, in general, the manuka honeys were the most effective at inhibiting growth, followed by the manuka-kanuka blends and then the kanuka honeys. However, the response of bacteria in the presence of sub-inhibitory concentrations of these different honeys varied with bacterial species, with each having a unique growth and morphological response. *P. aeruginosa* was very different to the other three bacteria in being both less sensitive overall and in having a similar response to the different honey types.

### High-throughput Analysis of Growth Dynamics Reveals that MGO in Honey Extends the Duration of Lag Phase

A high-throughput approach was used to assess the growth and morphological effects of a large number of natural honeys on multiple organisms. This approach is novel in honey studies and was employed here to address the challenge of assessing multiple parameters in a complex natural product. This system allowed us to explore the heterogeneous and variable composition of natural honey by analyzing large numbers of samples and control solutions, and showed the dynamic response of cell growth in response to the effects of honey toxicity. Such an approach may be useful in the study of other natural products where activity is modulated by various interacting factors.

Visual inspection of the resulting large number of growth curves revealed a distinctive dose-dependent extension of lag phase of growth when cultures of *B. subtilis*, *E. coli* and *S. aureus* were treated with manuka honey. This growth behavior was also observed when MGO alone was added to these bacterial cultures (Fig. 1), and is consistent with a previous study where *E. coli* was subjected to MGO treatment [29]. Lag-phase extension was not seen for clover or pure kanuka honeys; in these growth was either unaffected or was completely inhibited, and there was no evidence for dose-dependent recovery over time (Fig. S1). Thus the extended duration of lag phase is presumed to be largely or completely due to MGO and is likely to be unique to honey derived from manuka and other *Leptospermum* species.

### Growth and Morphology of Different Bacteria are Affected by Honey in Markedly Different Ways

The dynamics of growth in the presence of the different honey types was relatively similar for *B. subtilis, E. coli* and *S. aureus* but differed markedly in *P. aeruginosa* (Figs. 1 and 2). The extended duration of lag phase and the eventual resumption of logarithmic growth in the presence of MGO likely reflect induction of the glyoxylase system used to detoxify MGO [30]. All organisms produce MGO, which appears to be important in allowing them to regulate growth and maintain carbon flux as their environment changes [31]–[32]. However, as MGO is toxic, cells detoxify this compound to D-lactate using two metalloenzymes, GlxI and GlxII (Cooper, 1984). The ability of *P. aeruginosa* to grow in the presence of higher MGO levels than the other bacteria may reflect more efficient detoxification of MGO; a suggestion supported by the discovery, through genome sequencing, that *P. aeruginosa* is unique among eubacteria in its possession of three (rather than one) fully functional GlxI homologs [33].

To date, few microscopy studies have been performed to identify morphological changes to bacterial cells treated with honey, and none have used high-throughput phase-contrast and fluorescence microscopy that allows a large number of cells to be imaged and measured rapidly. We observed bacterial cell length changes in all organisms treated with manuka (high-MGO) honey. This is caused by an adjustment to the frequency of cell division relative to growth rate, often due to a change in nutritional state, such that division occurs at a different cell length to untreated cells [34]. Condensed DNA was also observed in a significant proportion of *B. subtilis* and *S. aureus* cells treated specifically with manuka honey. This could be a consequence of inhibition of initiation of DNA replication [35], [36]; a suggestion consistent with previous studies demonstrating that MGO alone inhibits this phase of DNA replication in bacterial cells [37]. Treatment with honey that contained the highest level of hydrogen peroxide (MK1) caused significant changes to the morphology of *B. subtilis* cells, including a dispersed appearance of the DNA. This could reflect a degree of DNA degradation due to hydrogen peroxide in the honey causing oxidative DNA damage [38].

With the exception of *B. subtilis,* the number and severity of morphological changes do not link clearly to the level of MGO or hydrogen peroxide in the honey, or to the effectiveness of the honey to inhibit growth. This is not entirely unexpected since cell morphology often reflects a response to changes in the environment that allows the organism to adapt to that environment without having to change its rate of growth. Different organisms do this differently when faced with a variety of nutritional and environmental conditions, such as oxidative or nutrient stress. This might reflect, at least in part, the degree of variation of the environment that these organisms inhabit [39]. We therefore speculate that the differences in morphology that we observe in response to a particular honey reflect species-specific differences in the regulatory circuits that coordinate growth with cellular physiology.

### MGO and Hydrogen Peroxide Production cannot Account for All Activity Present in Manuka, Kanuka and Clover Honey

Commensurate with previous studies [8], [15], [40], [41], we found that even when the peroxide activity was neutralized with catalase and there were negligible levels of MGO present, honey could inhibit bacterial growth. Even clover honey, with only trace levels of MGO and hydrogen peroxide, had variable effects on the four bacteria that in most cases could not be attributed to sugar alone. These observations are in line with previous studies suggesting that the presence of additional antibacterial components that may be directly active or may modulate the activity of the dominant active components [8], [40], [41]. These additional components may include: (i) phenolics derived from the floral source [19]; (ii) bee-derived antimicrobial peptides (although note that bee defensin-1, an antibacterial component of Revamil honey [8], could not be identified in manuka or kanuka honeys) [23] [42]; and (iii) as yet undefined synergistic compounds identified in other studies, including transition metals [38], [43], [44].

### Clinical Applications of Antibacterial Honey

The range of effects induced by the different honeys in the bacterial species tested reflects a diversity of responses that could be expected by bacteria present in chronic wounds. Our findings here have important implications for the clinical application of honey in the treatment of these wounds. First, sub-inhibitory concentrations of MGO may be neutralized by bacteria which then resume normal growth, thus any honey formulation should contain sufficient active honey to sustain inhibition. Second, honey without significant levels of MGO or hydrogen peroxide, such as clover honey, may be able to inhibit some bacteria but is not broad-spectrum and is therefore not recommended for infected wounds where multiple species may be present. Third, MGO at 600 mg/kg honey achieves almost as much inhibition as much higher concentrations, and increasing MGO above this threshold may not result in a more effective honey. And finally, in honey containing both MGO and hydrogen peroxide, MGO provides an over-riding activity and if this level is high enough, hydrogen peroxide does little to augment activity.

To date, more than 80 different microbial species, including bacteria and yeast pathogens known to infect wounds, have been shown to be inhibited by honey [22], [45], [46]. In the current study, the use of sub-inhibitory concentrations of honey has enabled us to examine the nature of honey inhibition, however these concentrations are well below those that would be used in a clinical situation, where whole honey is generally applied and complete and irreversible inhibition would be expected.

Emerging evidence from clinical studies suggests that honey is at least as effective as conventional treatments in healing wounds, particularly in very refractory cases such as in diabetics, the elderly, and extensively burned patients [47], [48], but more clinical data are necessary for robust statistical appraisal [49]. Here, we have demonstrated the potency of natural honey as an antimicrobial wound dressing, and that multiple effects arise from a variety of active compounds, which not only allows active honey to be uniquely broad in spectrum, but also reduces the potential for resistant microbial populations to evolve. Use of the full honey matrix is therefore recommended for the treatment of infected wounds. Understanding the complex nature of honeys and its effects on bacterial pathogens may eventually allow the development of specific blends with an optimal combination of antibacterial components, thus ensuring a highly effective and resilient antibacterial wound treatment option.

## Supporting Information

Figure S1 **The effect of New Zealand honey treatments on bacterial growth.** Growth curves of *B. subtilis* (001–032), *E. coli* (033–064), *S. aureus* (065–096) and *P. aeruginosa* (097–128) were treated with 10 different honeys (three manuka honeys, M1, M2, M3; four manuka/kanuka blended honeys, MK1, MK2, MK3, MK4; two kanuka honeys, K1, K2; and a clover honey, C) and a comprehensive range of controls, which included (i) a sugar solution comprising 45% of glucose, 48% of fructose and 1% of sucrose; (ii) honey plus catalase (1 mg/mL); (iii) a catalase-only control; (iv) three MGO solutions at starting concentrations matching that present in undiluted honeys M1, M2 and M3 (600, 1,000 & 1,500 mg/kg) and diluted the same as honey; v) a range of MGO concentrations plus catalase; and finally (vi) different MGO concentrations in the presence of both catalase and sugar solution at various concentrations (0% - as no honey control, 1%, 2%, 4%, 8%, 16% & 32% (w/v), represented by dark blue, light blue, green, pink, orange, purple and red color respectively). Optical density was recorded at 595 nm every h for 24 h. The optical density was then log-transformed and plotted against time using GraphPad Prism 5.0.
(PDF)

Figure S2 **Transformation of data obtained for bacterial growth with honey treatment.** Panel A illustrates the effect of various (1–32% (w/v)) concentrations of honey M3 on *E. coli* growth over 24 h as a simple log OD_595nm_ *versus* incubation time. The point at which 10% of the final OD_595nm_ is reached is shown by an ‘x’ on each growth curve. Panel B summarizes all the data from panel A as a simple relationship between honey concentration and the time it takes to reach 10% of the total OD_595nm_. A value of 24 hours on the y-axis denotes ‘no growth’.
(TIF)

Table S1 **Average cell length after different honey treatment (µm).** Cell lengths were not significantly affected by the honey treatments (*p>0.05*); all other values are significantly different (*p<0.05*); n ≥50. M3–4% manuka M3 (high-MGO) honey treatment. MK1–4% manuka-kanuka blended (high-hydrogen peroxide) honey treatment.
(DOCX)
