# Supplementary material for: The Effect of New Zealand Kanuka, Manuka and Clover Honeys on Bacterial Growth Dynamics and Cellular Morphology Varies According to the Species
Source: PLoS One. 2013 Feb 13;8(2):e55898. doi: 10.1371/journal.pone.0055898 (PMC3572166; doi:10.1371/journal.pone.0055898)
Supplement: Figure S1 — The effect of New Zealand honey treatments on bacterial growth. Growth curves of B. subtilis (001–032), E. coli (033–064), S. aureus (065–096) and P. aeruginosa (097–128) were treated with 10 different honeys (three manuka honeys, M1, M2, M3; four manuka/kanuka blended honeys, MK1, MK2, MK3, MK4; two kanuka honeys, K1, K2; and a clover honey, C) and a comprehensive range of controls, which included (i) a sugar solution comprising 45% of glucose, 48% of fructose and 1% of sucrose; (ii) honey plus catalase (1 mg/mL); (iii) a catalase-only control; (iv) three MGO solutions at starting concentrations matching that present in undiluted honeys M1, M2 and M3 (600, 1,000 & 1,500 mg/kg) and diluted the same as honey; v) a range of MGO concentrations plus catalase; and finally (vi) different MGO concentrations in the presence of both catalase and sugar solution at various concentrations (0% - as no honey control, 1%, 2%, 4%, 8%, 16% & 32% (w/v), represented by dark blue, light blue, green, pink, orange, purple and red color respectively). Optical density was recorded at 595 nm every h for 24 h. The optical density was then log-transformed and plotted against time using GraphPad Prism 5.0. (PDF) [file pone.0055898.s001.pdf]

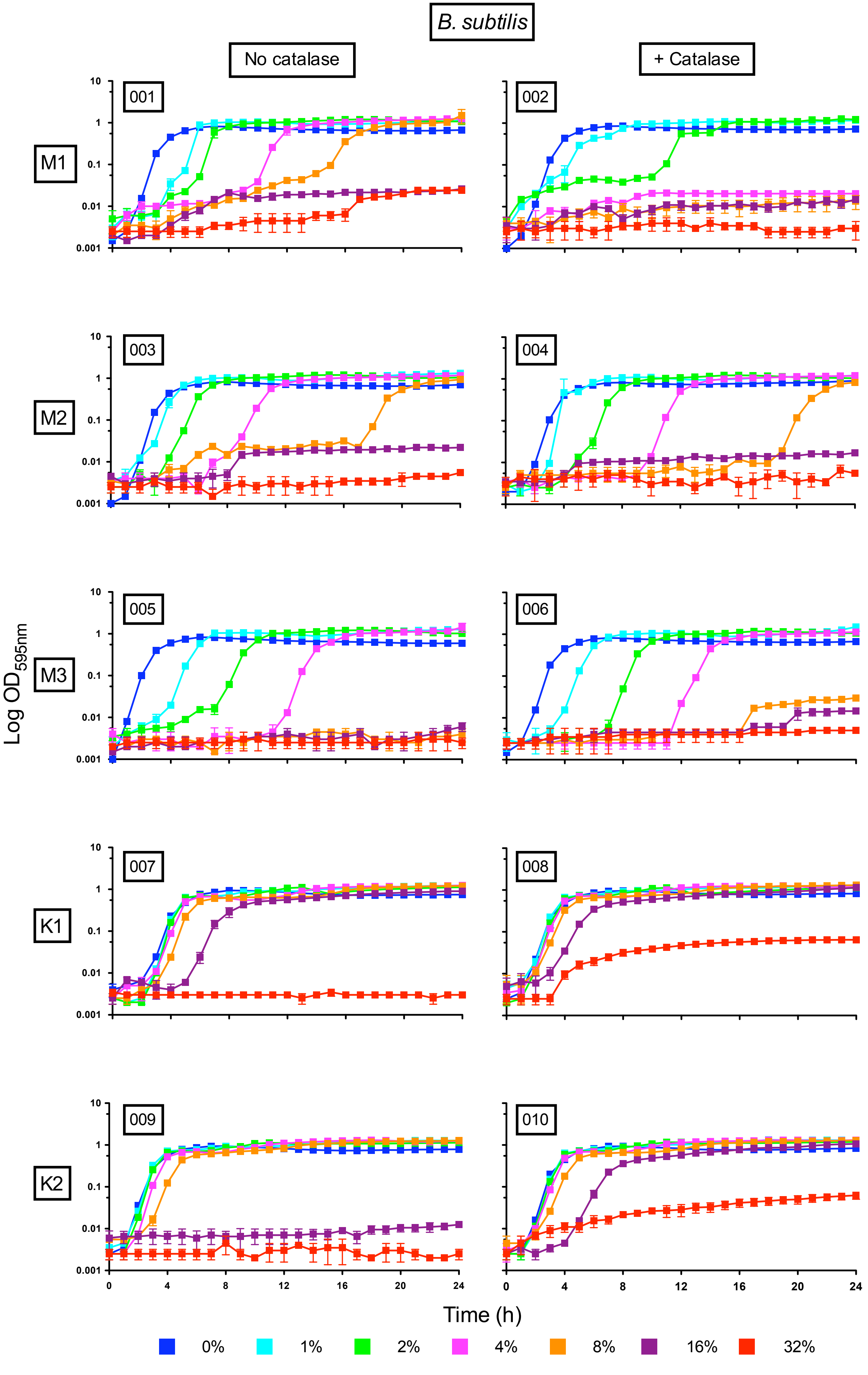

B. subtilis
No catalase
+ Catalase
M1
M2
M3
K1
K2
001
002
003
004
005
006
007
008
009
010
Log OD_{595nm}
Time (h)
0%
1%
2%
4%
8%
16%
32%

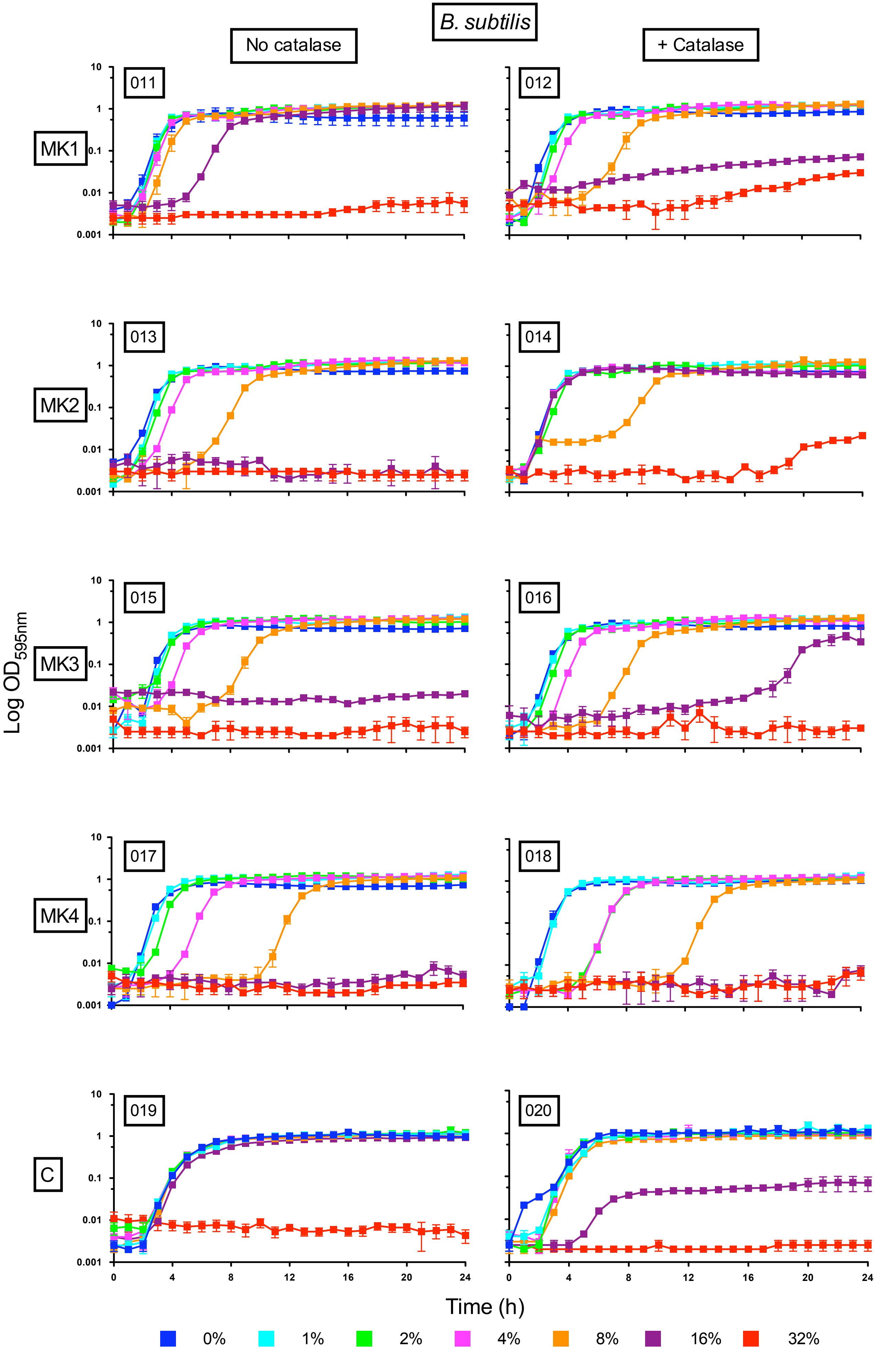
B. subtilis
No catalase
+ Catalase
MK1
MK2
MK3
MK4
C
011
012
013
014
015
016
017
018
019
020
Log OD595nm
10
1
0.1
0.01
0.001
0
4
8
12
16
20
24
Time (h)
0%
1%
2%
4%
8%
16%
32%

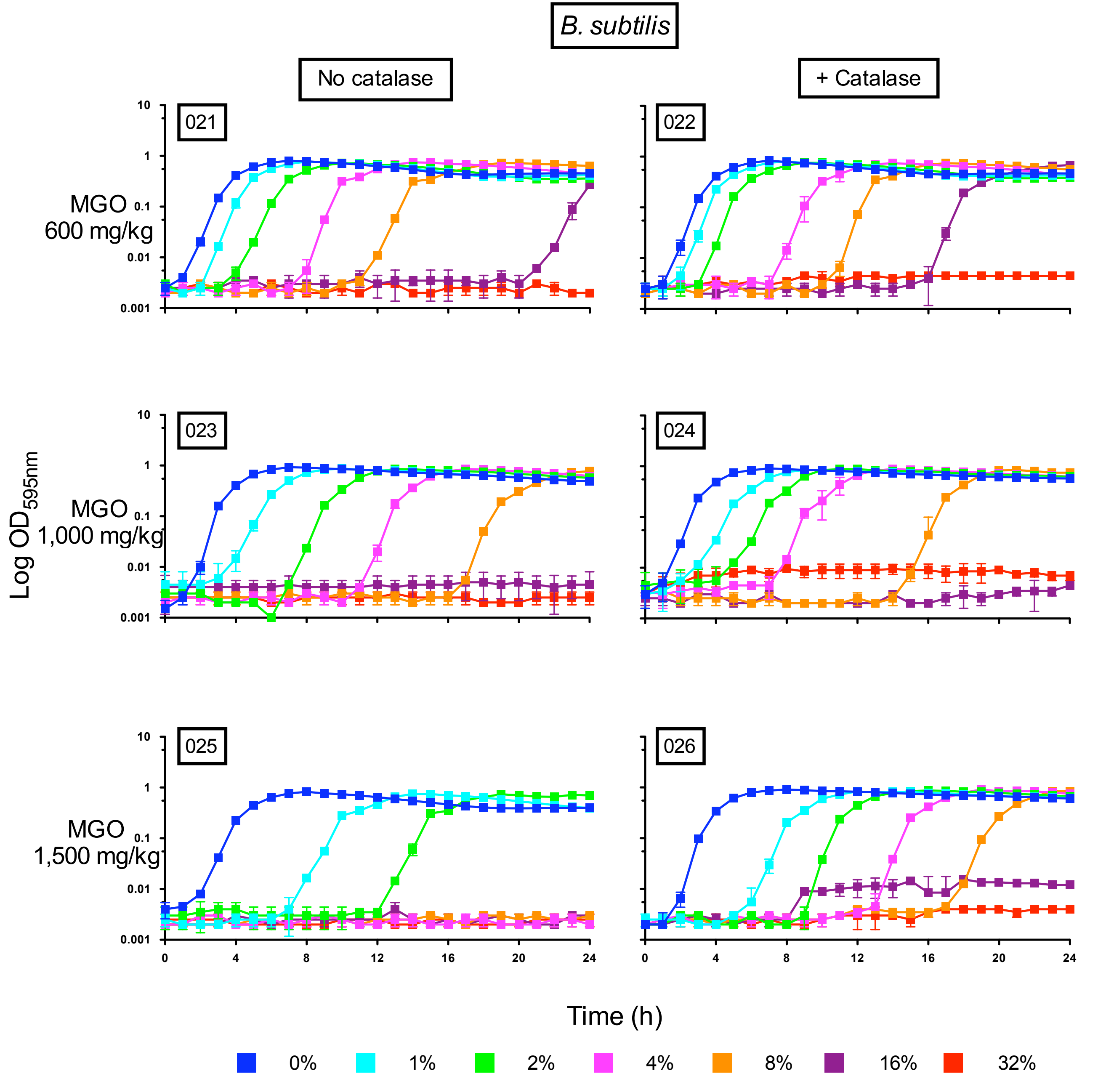

B. subtilis
No catalase
+ Catalase
021
022
023
024
025
026
MGO 600 mg/kg
MGO 1,000 mg/kg
MGO 1,500 mg/kg
Log OD595nm
Time (h)
0%
1%
2%
4%
8%
16%
32%

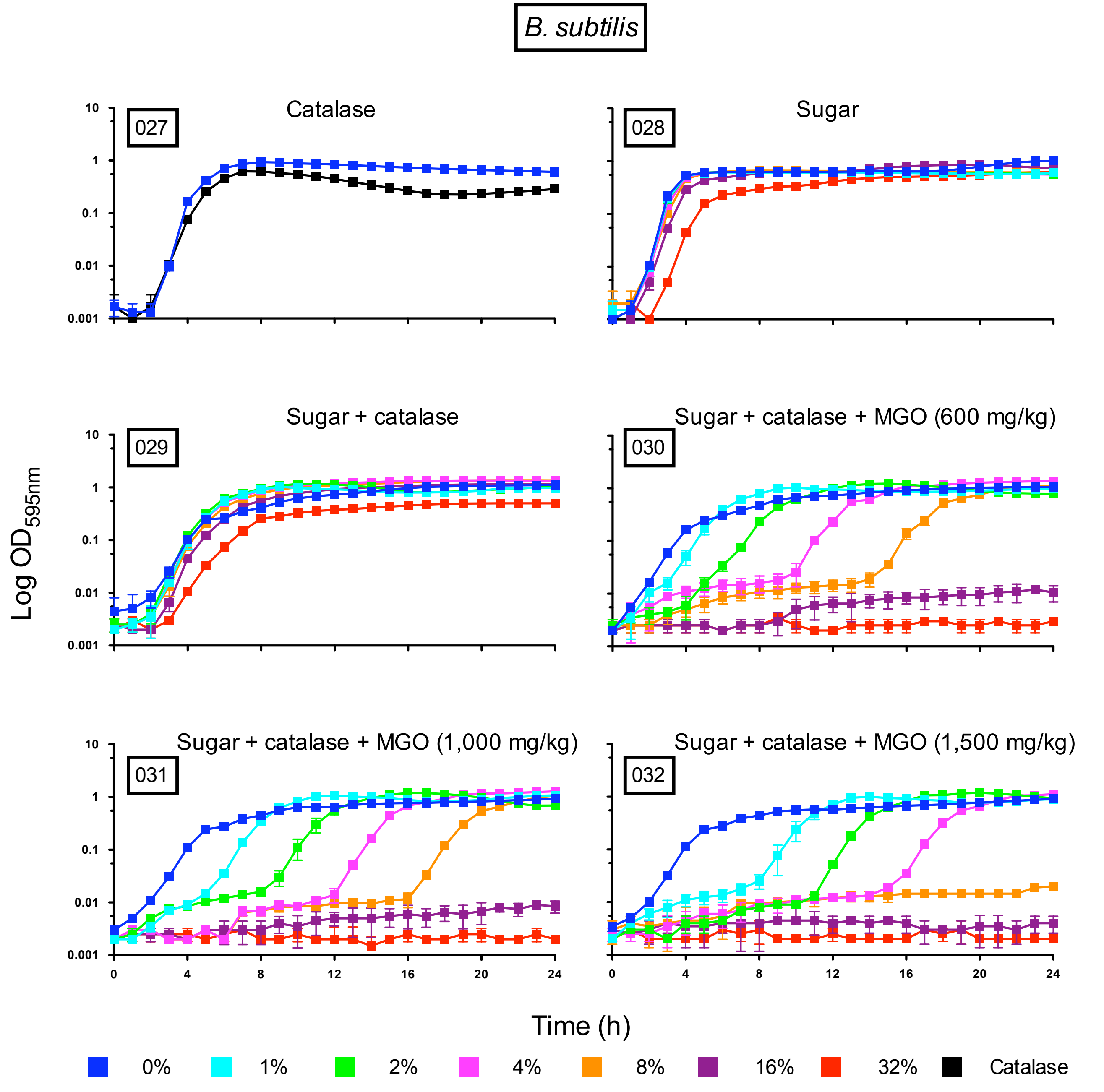

B. subtilis
Catalase
027
Sugar
028
Sugar + catalase
029
Sugar + catalase + MGO (600 mg/kg)
030
Sugar + catalase + MGO (1,000 mg/kg)
031
Sugar + catalase + MGO (1,500 mg/kg)
032
10
1
0.1
0.01
0.001
0
4
8
12
16
20
24
Log OD_{595nm}
Time (h)
0%
1%
2%
4%
8%
16%
32%
Catalase

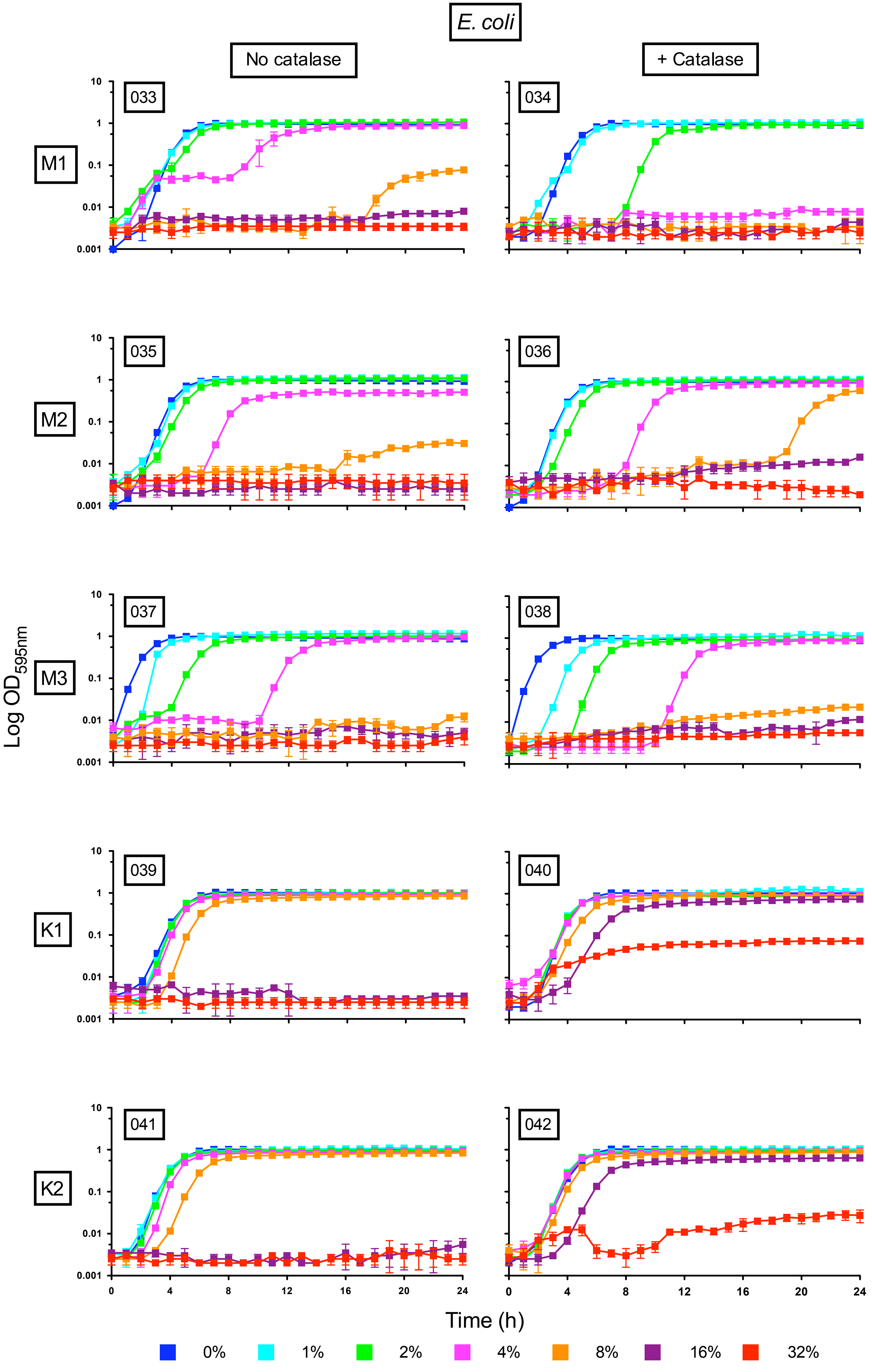

E. coli
No catalase
+ Catalase
M1
M2
M3
K1
K2
033
034
035
036
037
038
039
040
041
042
Log OD595nm
10
1
0.1
0.01
0.001
0
4
8
12
16
20
24
Time (h)
0%
1%
2%
4%
8%
16%
32%

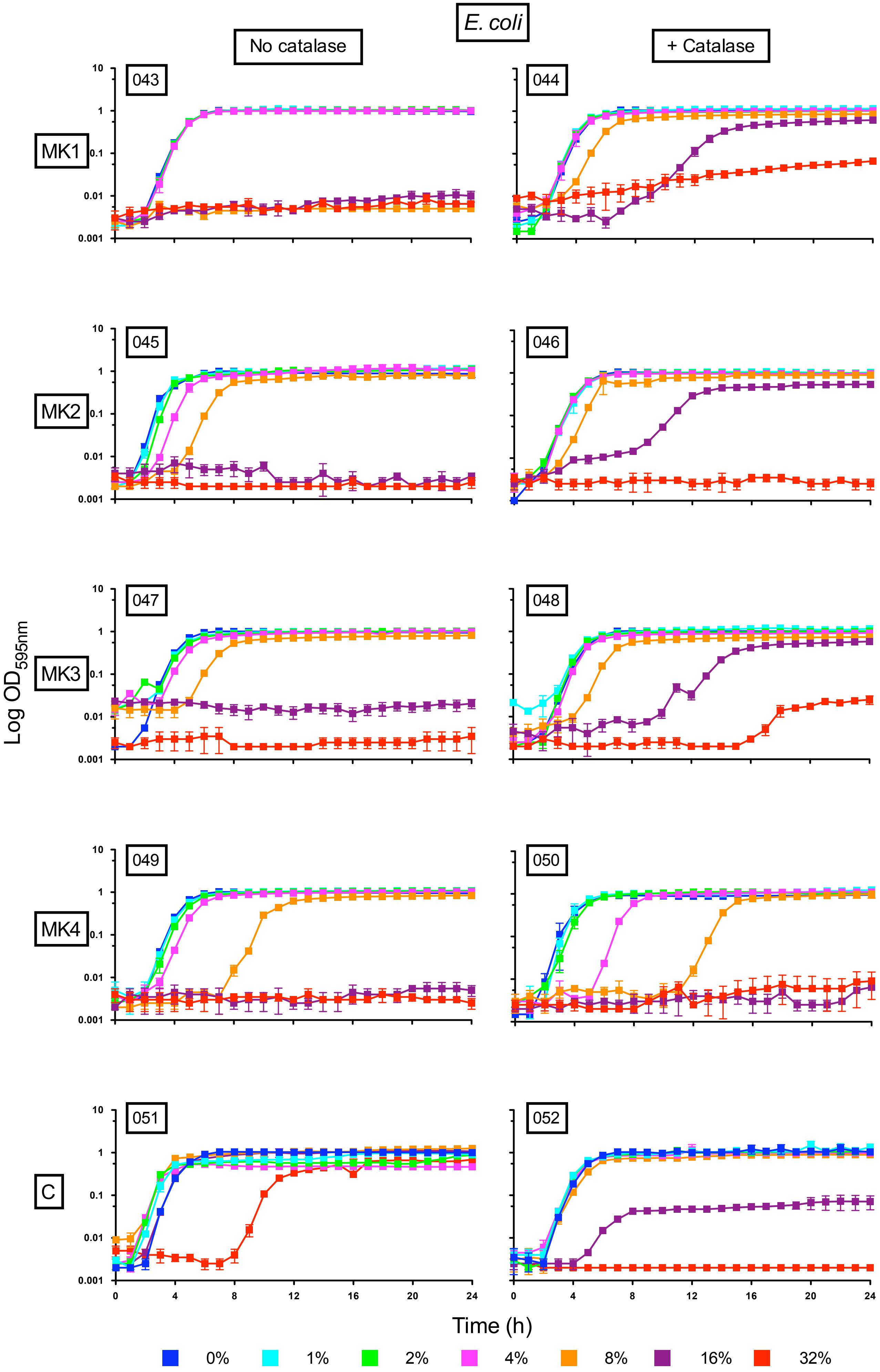
E. coli
No catalase
+ Catalase
MK1
MK2
MK3
MK4
C
043
044
045
046
047
048
049
050
051
052
Log OD_{595nm}
Time (h)
0%
1%
2%
4%
8%
16%
32%

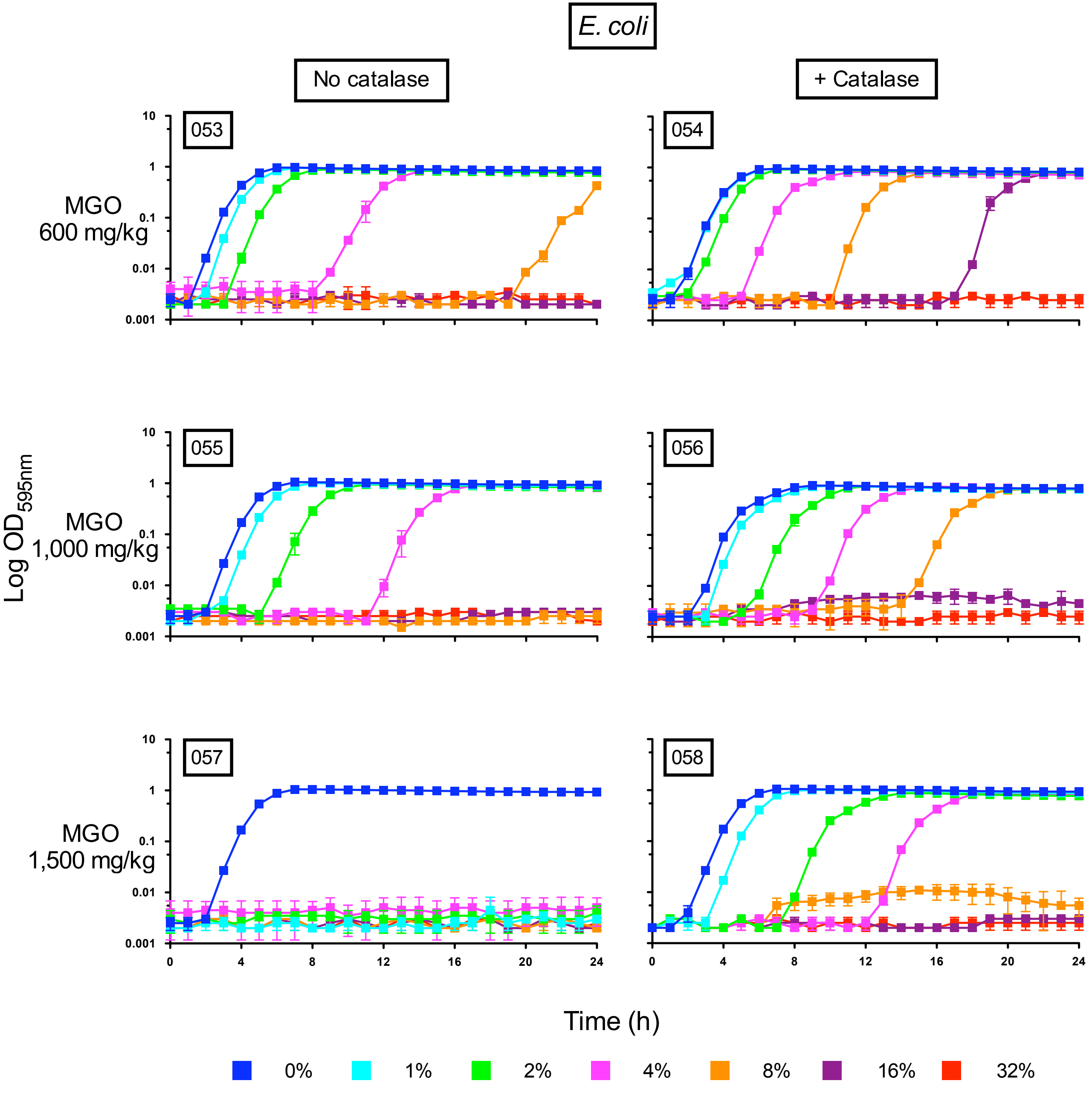

E. coli
No catalase
+ Catalase
053
054
055
056
057
058
MGO 600 mg/kg
MGO 1,000 mg/kg
MGO 1,500 mg/kg
Log OD_{595nm}
Time (h)
0%
1%
2%
4%
8%
16%
32%

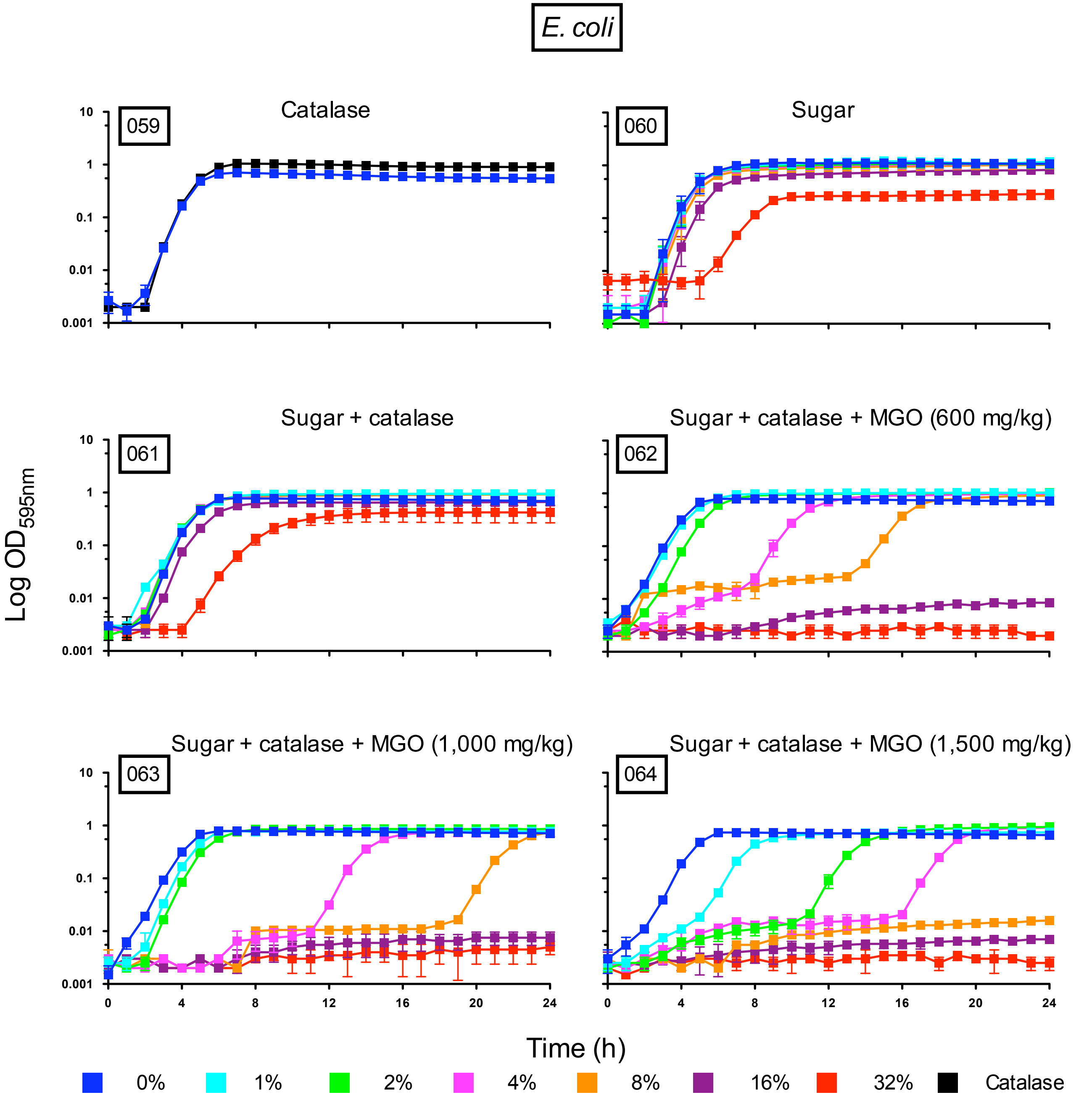

E. coli
Catalase
059
Sugar
060
Sugar + catalase
061
Sugar + catalase + MGO (600 mg/kg)
062
Sugar + catalase + MGO (1,000 mg/kg)
063
Sugar + catalase + MGO (1,500 mg/kg)
064
Log OD595nm
10
1
0.1
0.01
0.001
0
4
8
12
16
20
24
Time (h)
0%
1%
2%
4%
8%
16%
32%
Catalase

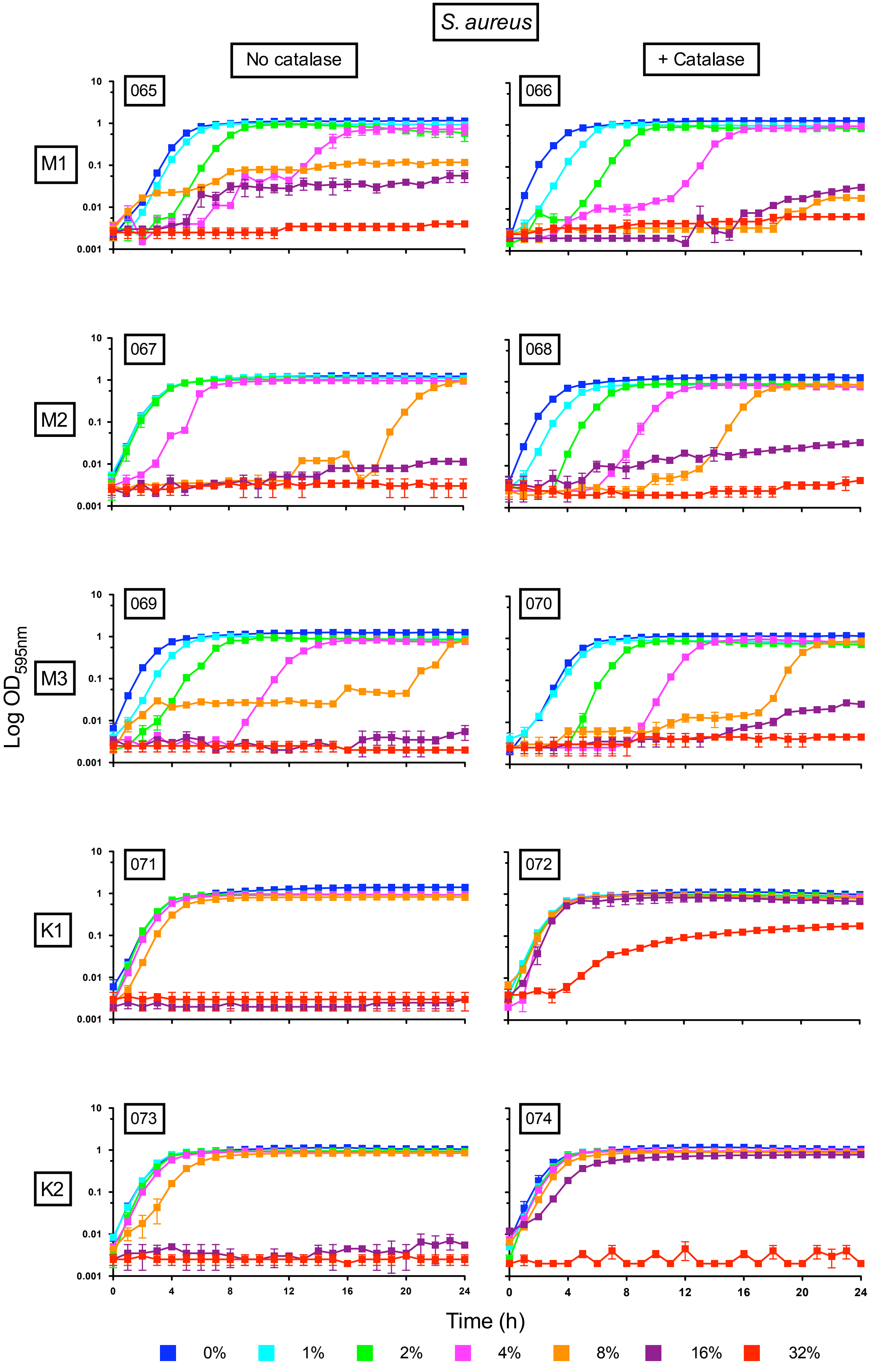

S. aureus
No catalase
+ Catalase
065
066
067
068
069
070
071
072
073
074
M1
M2
M3
K1
K2
Log OD595nm
10
1
0.1
0.01
0.001
0
4
8
12
16
20
24
Time (h)
0%
1%
2%
4%
8%
16%
32%

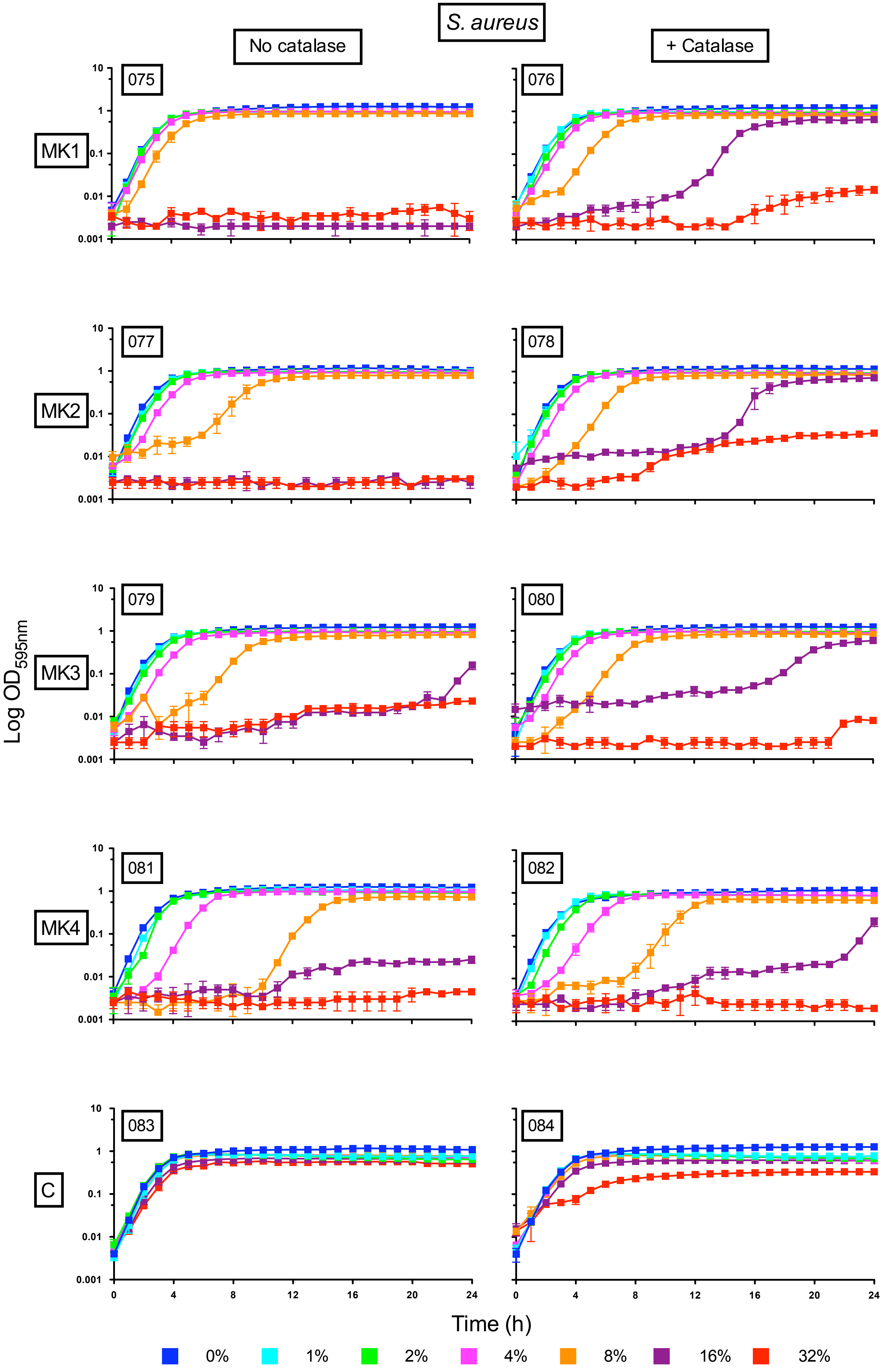

S. aureus
No catalase
+ Catalase
MK1
MK2
MK3
MK4
C
075
076
077
078
079
080
081
082
083
084
Log OD595nm
10
1
0.1
0.01
0.001
0
4
8
12
16
20
24
Time (h)
0%
1%
2%
4%
8%
16%
32%

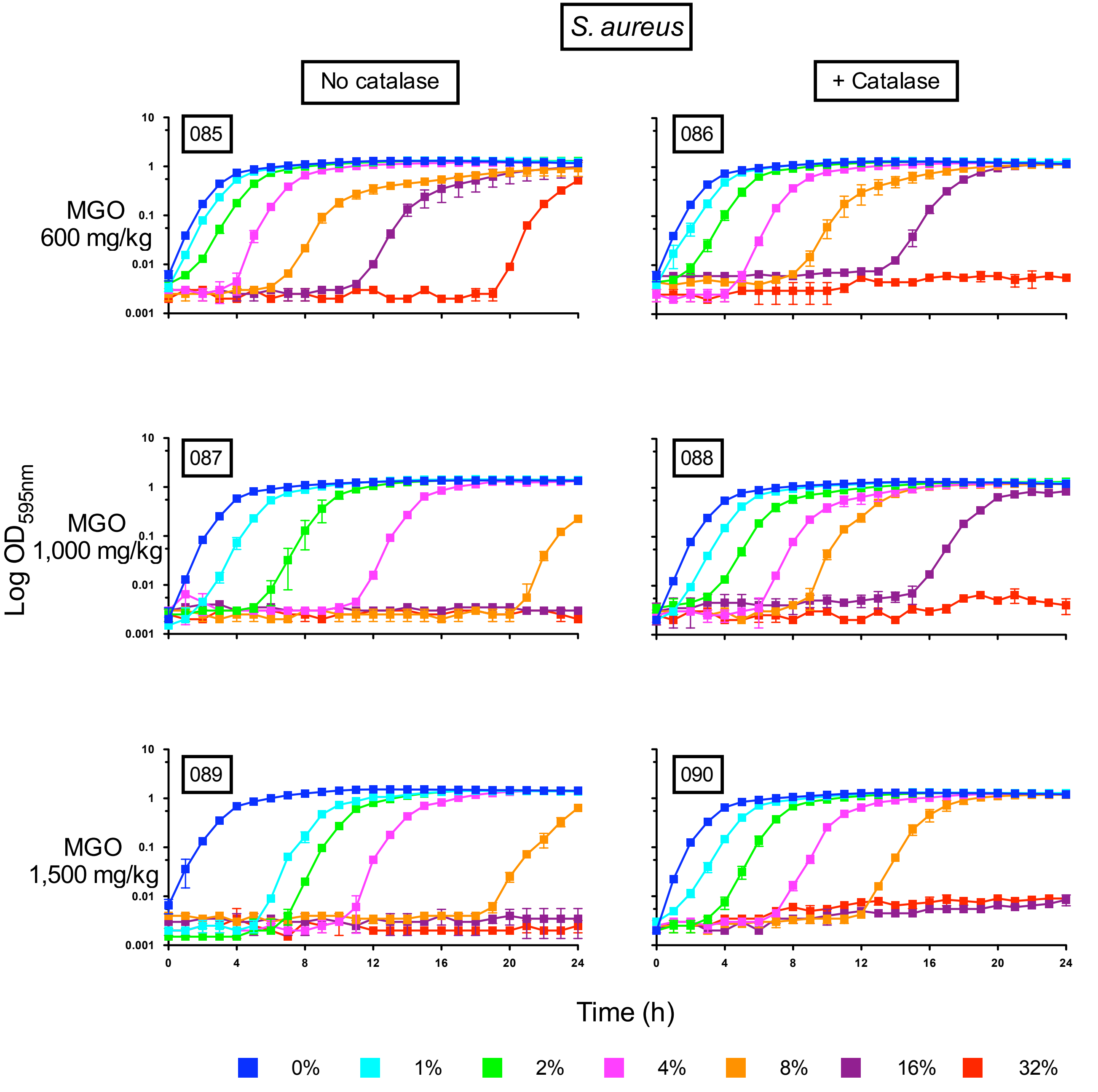
S. aureus
No catalase
+ Catalase
085
086
087
088
089
090
MGO 600 mg/kg
MGO 1,000 mg/kg
MGO 1,500 mg/kg
Log OD_{595nm}
10
1
0.1
0.01
0.001
0
4
8
12
16
20
24
Time (h)
0%
1%
2%
4%
8%
16%
32%

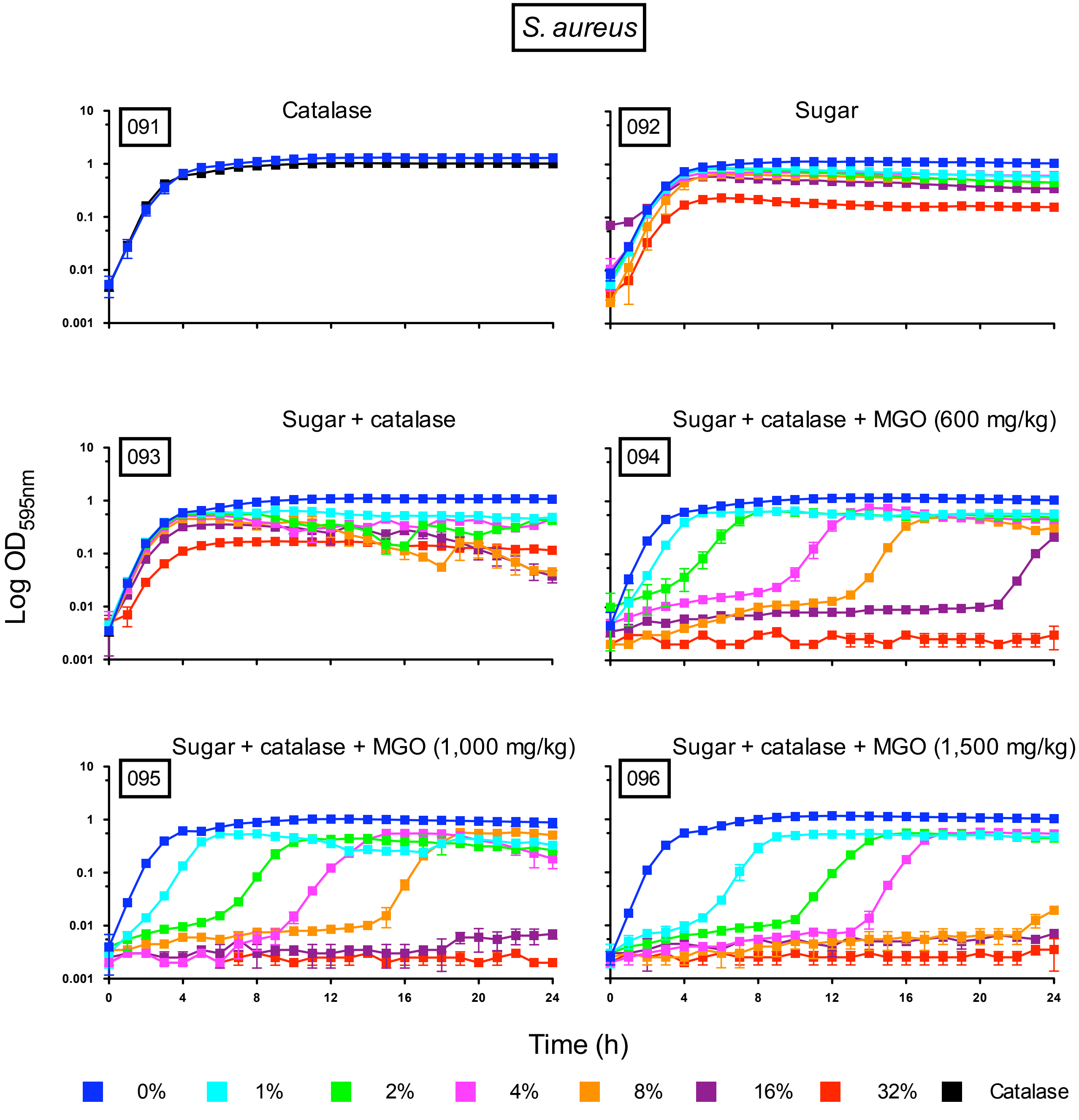
S. aureus
091
Catalase
092
Sugar
093
Sugar + catalase
094
Sugar + catalase + MGO (600 mg/kg)
095
Sugar + catalase + MGO (1,000 mg/kg)
096
Sugar + catalase + MGO (1,500 mg/kg)
10
1
0.1
0.01
0.001
0
4
8
12
16
20
24
Log OD_{595nm}
Time (h)
0%
1%
2%
4%
8%
16%
32%
Catalase

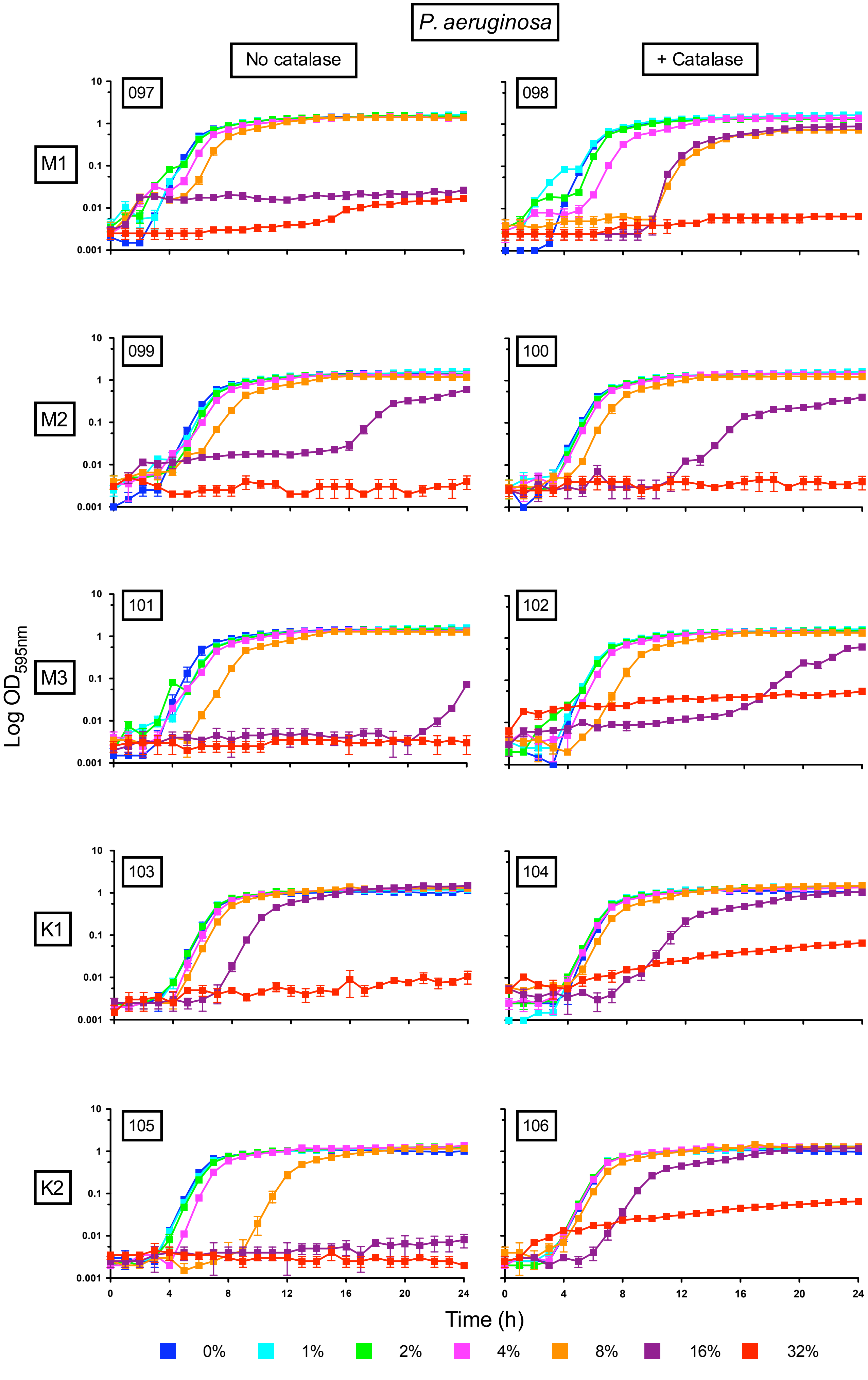

P. aeruginosa
No catalase
+ Catalase
097
098
099
100
101
102
103
104
105
106
M1
M2
M3
K1
K2
Log OD595nm
Time (h)
10
1
0.1
0.01
0.001
0
4
8
12
16
20
24
0%
1%
2%
4%
8%
16%
32%

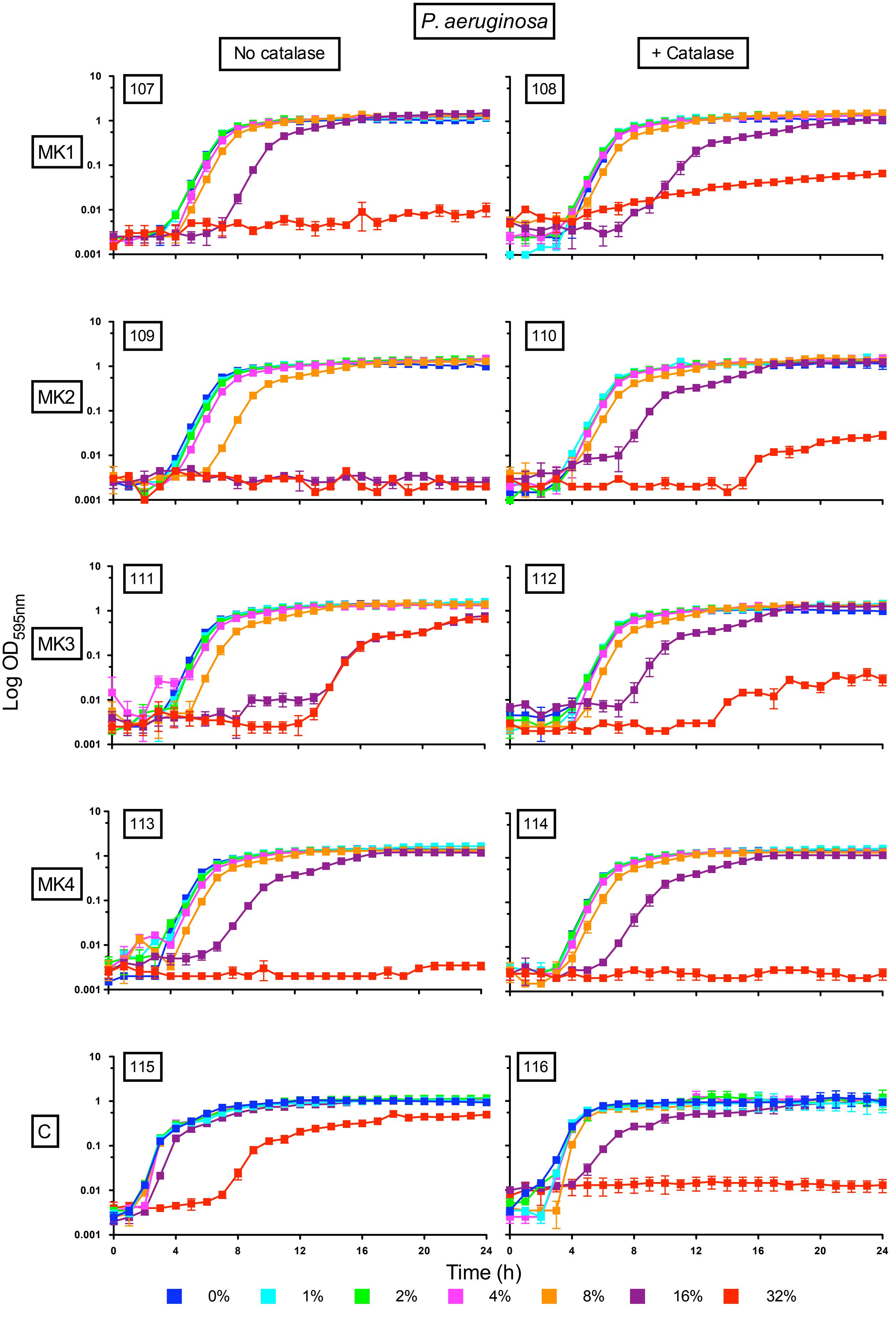

P. aeruginosa
No catalase
+ Catalase
107
108
109
110
111
112
113
114
115
116
MK1
MK2
MK3
MK4
C
Log OD595nm
10
1
0.1
0.01
0.001
0
4
8
12
16
20
24
Time (h)
0%
1%
2%
4%
8%
16%
32%

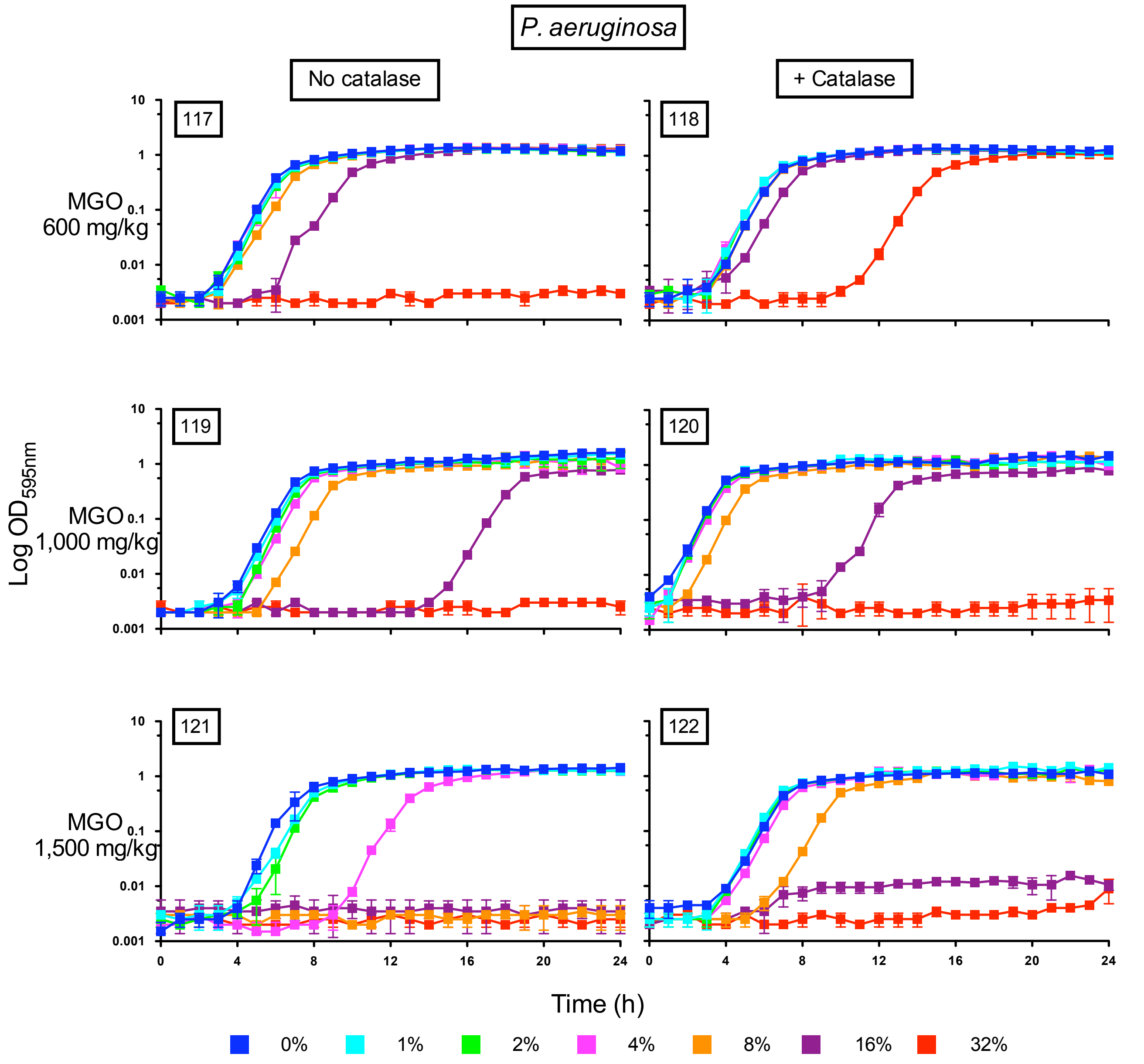
P. aeruginosa
No catalase
+ Catalase
117
118
119
120
121
122
MGO 600 mg/kg
MGO 1,000 mg/kg
MGO 1,500 mg/kg
Log OD595nm
10
1
0.1
0.01
0.001
0
4
8
12
16
20
24
Time (h)
0%
1%
2%
4%
8%
16%
32%

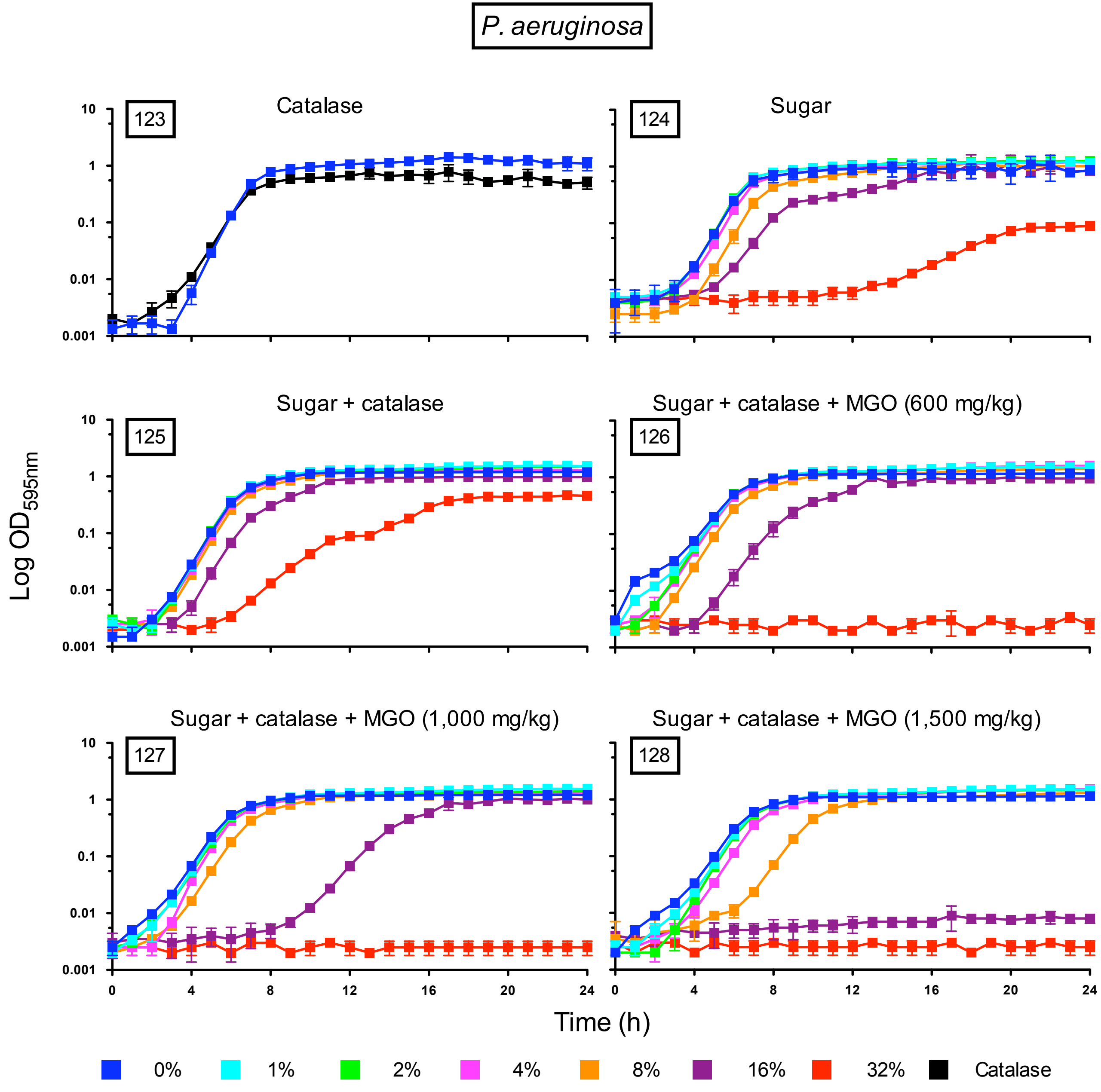

P. aeruginosa
123
Catalase
124
Sugar
125
Sugar + catalase
126
Sugar + catalase + MGO (600 mg/kg)
127
Sugar + catalase + MGO (1,000 mg/kg)
128
Sugar + catalase + MGO (1,500 mg/kg)
Log OD595nm
10
1
0.1
0.01
0.001
0
4
8
12
16
20
24
Time (h)
0%
1%
2%
4%
8%
16%
32%
Catalase
