# Supplementary figures and images for: The Effect of New Zealand Kanuka, Manuka and Clover Honeys on Bacterial Growth Dynamics and Cellular Morphology Varies According to the Species
Source: PLoS One. 2013 Feb 13;8(2):e55898. doi: 10.1371/journal.pone.0055898 (PMC3572166; doi:10.1371/journal.pone.0055898)

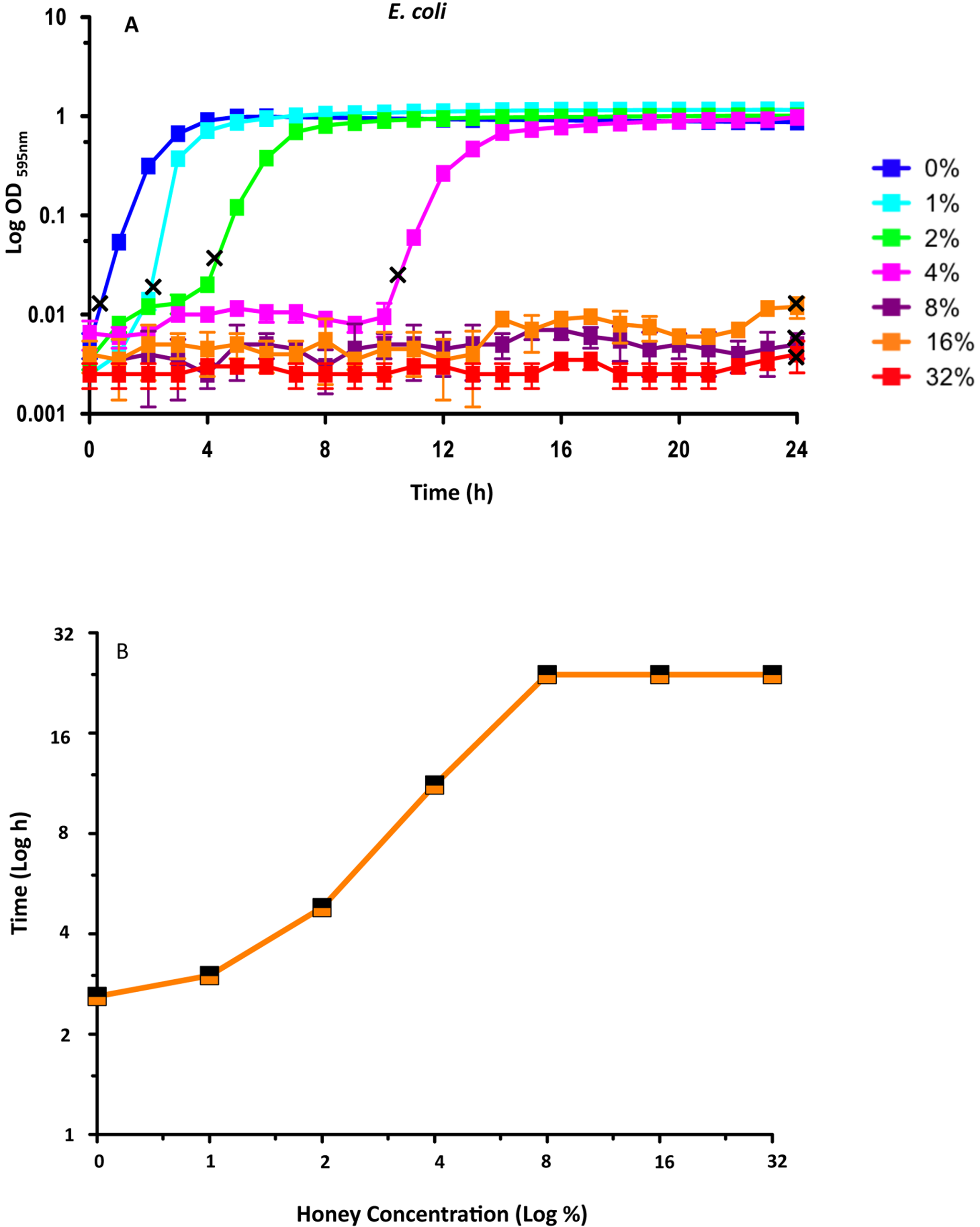

Supplement: Figure S2 — Transformation of data obtained for bacterial growth with honey treatment. Panel A illustrates the effect of various (1–32% (w/v)) concentrations of honey M3 on E. coli growth over 24 h as a simple log OD595nm versus incubation time. The point at which 10% of the final OD595nm is reached is shown by an ‘x’ on each growth curve. Panel B summarizes all the data from panel A as a simple relationship between honey concentration and the time it takes to reach 10% of the total OD595nm. A value of 24 hours on the y-axis denotes ‘no growth’. (TIF) [file pone.0055898.s002.tif]
