# Supplementary material for: The Effect of New Zealand Kanuka, Manuka and Clover Honeys on Bacterial Growth Dynamics and Cellular Morphology Varies According to the Species
Source: PLoS One. 2013 Feb 13;8(2):e55898. doi: 10.1371/journal.pone.0055898 (PMC3572166; doi:10.1371/journal.pone.0055898)
Supplement: Table S1 — Average cell length after different honey treatment (µm). Cell lengths were not significantly affected by the honey treatments (p>0.05); all other values are significantly different (p<0.05); n ≥50. M3–4% manuka M3 (high-MGO) honey treatment. MK1–4% manuka-kanuka blended (high-hydrogen peroxide) honey treatment. (DOCX) [file pone.0055898.s003.docx]

**Table S1 – Average cell length after different honey treatment (μm)**

|  | ***B. subtilis*** | | | ***E. coli*** | | | ***S. aureus*** | | | ***P. aeruginosa*** | | |
| --- | --- | --- | --- | --- | --- | --- | --- | --- | --- | --- | --- | --- |
|  | **0%** | **M3** | **MK1** | **0%** | **M3** | **MK1** | **0%** | **M3** | **MK1** | **0%** | **M3** | **MK1** |
| **Lag** | 4.4±1.8 | 2.5±0.6 | 3.8±0.8 | 3.3±1.1 | 1.8±0.3 | 3.4±1.1^^^ | 1.2±0.2 | 1.0±0.2 | 1.1±0.2 | 2.5±0.6 | 2.7±0.5 | 2.4±0.6^^^ |
| **Mid-log** | 4.2±1.1 | 3.0±0.9 | 3.0±0.7 | 2.6±1.5 | 4.1±3.6 | 2.4±2.2^^^ | 1.1±0.1 | 0.9±0.2 | 1.1±0.2^^^ | 2.5±0.5 | 2.3±0.5 | 1.6±0.4 |

**^^^** Cell lengths were not significantly affected by the honey treatments (*p>0.05*); all other values are significantly different (*p<0.05*); n ≥ 50.

M3 – 4% manuka M3 (high-MGO) honey treatment

MK1 – 4% manuka-kanuka blended (high-hydrogen peroxide) honey treatment
